# Effects of single and repeated drought on soil microarthropods in a semi-arid ecosystem depend more on timing and duration than drought severity

**DOI:** 10.1371/journal.pone.0219975

**Published:** 2019-07-18

**Authors:** Norbert Flórián, Márta Ladányi, András Ittzés, György Kröel-Dulay, Gábor Ónodi, Márton Mucsi, Tibor Szili-Kovács, Veronika Gergócs, László Dányi, Miklós Dombos

**Affiliations:** 1 Institute for Soil Sciences and Agricultural Chemistry, Centre for Agricultural Research, Hungarian Academy of Sciences, Budapest, Hungary; 2 Department of Biometrics and Agricultural Informatics, Faculty of Horticultural Science, Szent István University, Budapest, Hungary; 3 Institute of Ecology and Botany, Centre for Ecological Research, Hungarian Academy of Sciences, Vácrátót, Hungary; 4 Department of Zoology, Hungarian Natural History Museum, Budapest, Hungary; Feroze Gandhi Degree College, INDIA

## Abstract

Soil moisture is one of the most important factors affecting soil biota. In arid and semi-arid ecosystems, soil mesofauna is adapted to temporary drought events, but, until now, we have had a limited understanding of the impacts of the different magnitudes and frequencies of drought predicted to occur according to future climate change scenarios. The present study focuses on how springtails and mites respond to simulated repeated drought events of different magnitudes in a field experiment in a Hungarian semi-arid sand steppe. Changes in soil arthropod activities were monitored with soil trapping over two years in a sandy soil. In the first year (2014), we applied an extreme drought pretreatment, and in the consecutive year, we applied less devastating treatments (severe drought, moderate drought, water addition) to these sites. In the first year, the extreme drought pretreatment tended to have a negative effect (either significantly or not significantly) on the capture of all Collembola groups, whereas all mite groups increased in activity density. However, in the consecutive year, between the extreme drought and control treatments, we only detected differences in soil microbial biomass. In the cases of severe drought, moderate drought and water addition, we did not find considerable changes across the microarthropods, except in the case of epedaphic Collembola. In the cases of the water addition and drought treatments, the duration and timing of the manipulation seemed to be more important for soil mesofauna than their severity (i.e., the level of soil moisture decrease). We suggest that in these extreme habitats, soil mesofauna are able to survive extreme conditions, and their populations recover rapidly, but they may not be able to cope with very long drought periods.

## Introduction

Climate change is one of the most current issues in soil protection. Global temperature is rising, and a 2.5–3°C summer temperature increase is predicted to occur by 2021–2040 relative to the 1981–2000 period [1,2]. Different climate change factors act differently among climate zones: more warming is predicted in the northern region, whereas in addition to an elevated level of CO_2_ [3], drought seems to have high importance in temperate areas. Due to climate change, an increased intensity and frequency of extreme events, such as severe droughts, heavy rains, and heat waves, is expected [4,5].

Soil mesofauna (mostly microarthropods of 0.2–4 mm in body size) occupy a central position in the soil food web eg. [6], especially in semi-arid areas, where earthworms are rare [7]. Among them, springtails and oribatid mites, as the most abundant groups, can influence the process and regulate the speed of decomposition and exert strong feedback on plants [8–10]. They are also often used as bioindicators because of their rapid reaction to environmental shifts [11,12]. Moreover, soil mesofauna have a small activity range; therefore, compared to macroinvertebrates, they are better suited for small-scale experiments [13].

Soil moisture is one of the most important factors affecting soil communities [14–19]. Drought can be separated from warming effects [20] and has direct or indirect effects on soil microarthropods [21–23]. Desiccation, through dehydration, has a direct effect on mesofauna, influencing their survival and inducing physiological and behavioural changes eg. [24]. However, indirect effects of drought seem to be more important in soil ecosystems. Drought may decrease the decomposition rate [25], alter soil characteristics [26] and change the quantity and quality of plant litter, influencing bottom-up effects [27]. Changes in the decomposition rate and pathway also influence vegetation structure and productivity through soil animal communities [21,22].

The precipitation regime of a region may determine the biological responses to drought events [28]. Different levels of soil moisture caused by rain pulses are reported to influence the activity patterns of macroarthropods, but our knowledge regarding the effects on microarthropods is limited [29]. The reaction of soil mesofauna to drought can be rapid, but their population sizes may recover over a short time [30]. Most soil-dwelling microarthropods live in a stable environment. However, they can survive under dry conditions eg. [11,31], especially in arid and semi-arid areas, where drought occurs regularly for a certain period and with different intensities [23]. According to Nielsen and Ball [28], changes in the intensity and frequency of climate events influence the ecological functioning of soil invertebrates and may affect the carbon and nutrient pool of the soil. However, only a few studies have focused on alterations to the magnitude or frequency of precipitation in arid or semi-arid ecosystems [15,18,29]. In previous experiments, a high amount of precipitation increased the abundance of soil mesofauna [18], whereas a high frequency had no [15] or variable effects [29] depending on the animal group considered. Moreover, we have a limited understanding regarding how an extreme or altered intensity of repeated drought events affects soil communities and how rapidly their populations recover after drought shock.

In our field experiment, we investigated how an extreme event (extreme drought) and subsequent moderate changes in precipitation, in accordance with climate change scenarios, affect the soil microbial biomass and soil microarthropods in a semi-arid ecosystem. With a new sampling method, we measured the activity density (AD; i.e., the number of individuals in the traps derived from their abundance and activity [32]) and species richness of different soil microarthropod groups in sandy soils in a manner similar to that used for pitfall traps. The treatments included four different levels of drought: extreme (5 months) drought as a pretreatment in the first year and severe (2 months) drought, moderate (1 month) drought and water addition in the second year. In the second year, microbial biomass carbon (MBC) was also measured as an indicator of bottom-up effects on the soil mesofauna.

The main goal of this study was to reveal the effects of different levels of drought (extreme (5 months), severe (2 months), modest (1 month) and extra precipitation) on the activity density (AD) of soil microarthropods. We hypothesized that the different drought treatments (from modest to extreme) would negatively influence the AD of soil microarthropods, whereas for the extra-precipitation treatment, a positive response of soil fauna was hypothesized to occur. Finally, the agonistic or antagonistic effects of the different treatments on microarthropods were compared between the two years.

## Methods

### Study site

Our experiment took place in an open sand steppe (46°52’16.6”N, 19°25’17.7”E), near Fülöpháza in Kiskunság National Park, central Hungary (field permit number: 71.293-5-1/2012, Alsó-Tisza-vidéki Környezetvédelmi, Természetvédelmi és Vízügyi Felügyelőség). The vegetation is dominated by perennial grasses, *Festuca vaginata* and *Stipa borysthenica*. The study site has a sandy soil (calcaric arenosol) [33] with pH 7.8 and 1.2% silt, 1.5% clay, and 97.3% sand content. The area has a continental climate with a long-term mean annual precipitation of 500–550 mm and a mean monthly temperature of -1.8°C in January to 21°C in July [34].

For central Europe, regional climate change models predict warmer and drier summers and milder but wetter winters [35], causing severe droughts, especially in the sand dunes region in Hungary, called the Sand Ridge [36]. Extreme conditions are usual in sand steppes; thus, the biota of these ecosystems can cope with such extreme conditions up to a certain level. Over the last four decades, the water table has fallen 4–5 metres [37]; therefore, the area is threatened by desertification and is considered one of the most vulnerable parts of Hungary. Therefore, this area has the potential to be an important model ecosystem from both ecological and conservation perspectives. Sand steppes are water limited and are directly affected by precipitation changes.

### Experimental design

The study site is homogeneously covered by open sand steppe, but it shows some spatial variability in elevation, exposition and plant species dominance. Therefore, we selected six blocks, ca. 12 x 6 m in size, that were internally homogeneous in these factors. In each block, there were eight 3 x 3 m plots, with four plots being adjacent to one another and thus comprising a 6 x 6 m area and another four plots comprising another 6 x 6 m area, with the plots ca. 2 m apart (Figure A in S2 File). Within each block in the first year (2014), we applied an extreme drought pretreatment (two levels: extreme drought (X) vs. control (C)). In the consecutive year (2015), we applied mild precipitation at four levels: severe drought (S), moderate drought (M), control (C, ambient precipitation) and water addition (W) in both of the previously X- and C-treated plots. In this way, the two factors (i.e., extreme drought and mild precipitation change) were combined in a full factorial design, resulting in eight treatment combinations (CC, CS, CM, CW, XC, XS, XM, XW) with six replicates for a total of 48 study plots.

The extreme drought was simulated by excluding all rain from 24 April 2014 to 18 September 2014 by permanently covering these plots with transparent polyethylene roofs. The height of the roofs in all treatments varied from 80 to 100 cm depending on the topography. Severe drought was simulated by covering the respective plots for two months in 2015 (23 June to 25 August in 2015), while moderate drought was simulated by covering these plots for one month in 2015 (from 20 July to 25 August). Water addition was applied four times: 25 May, 22 June, 21 July and 25 August. We added a total of 98.5 mm of water (which is the mean value of two months of the mean summer precipitation in the area) in four approximately equal parts, imitating the amount of precipitation received during a thunderstorm. This amount was 18.8% of the ambient precipitation in that year. During water addition, we applied the collected rainwater with sprinklers from ca. 70 cm height. At the time of sprinkling, side-curtains were used to prevent irrigation water from falling into the neighbouring plots. While all treatments were applied to the 3 x 3 m plots, we designated the inner 2 x 2 m parts as the core area: all measurements were conducted there, and the outer 0.5 m zones were considered buffers.

### Environmental variables and biological sampling

#### Measurement of environmental variables

Soil humidity was measured at each plot in situ at a 0–30 cm soil depth with a Campbell CS616 soil moisture sensor. Soil temperature data were obtained from a 10 cm soil depth with a Jumo RTD (Pt100) temperature probe. These instruments provided data every 10 minutes, and mean values were calculated for each day (N = 365).

#### Effects of the treatments on soil moisture and temperature

The extreme drought treatment (X) was effective in 2014, as the soil humidity dropped markedly (Figure B in S2 File) during and after the treatment. We excluded 64.1% (523.5 mm) of the annual precipitation.

However, the extreme drought treatment had the opposite effect in 2015. During the vegetation period, the soil moisture was higher in the previously (in 2014) extreme drought-treated plots (XC) compared to the control plots (CC) (Figure B in S2 File). Concerning the effects of the second factor, in 2015, the soil moisture in the severe drought treatment (CS) declined to the values measured in the extreme drought plots in 2014 (XC) (Figure B in S2 File, Table 1). In 2015, 23.3% of the yearly precipitation (121.8 mm) was excluded from the severe drought treatment plots. In the moderate drought treatment (M), 18.2% of the yearly precipitation (95.4 mm) was excluded. In terms of soil moisture, we attained the same level of drought as in the S treatment but for a shorter period (one month) (Figure B in S2 File; Table 1). For the water addition treatment (W), we added extra precipitation (a total of 98.5 mm), which caused no increase in soil moisture over the long term, only immediately after sprinkling (Figure B in S2 File).

**Table 1:** Environmental variables.

	2014 C	2014 X	2015 C	2015 W	2015 M	2015 S
mean soil moisture (12 months)	5.60±0.1	4.19±0.1	5.06±0.1	5.01±0.1	4.66±0.1	4.63±0.1
mean soil moisture (August)	5.65±0.2	2.15±0.1	4.67±0.2	4.40±0.2	2.47±0.2	2.11±0.1
lowest soil moisture	3.0	2.1	2.4	2.5	2.1	2.0
mean soil temperature (12 months)	14.76±0.5	15.46±0.5	14.64±0.4	14.69±0.4	14.79±0.4	15.10±0.4
mean soil temperature (August)	24.79±0.4	26.91±0.4	26.75±0.4	26.67±0.4	28.43±0.4	29.16±0.4

Values of environmental variables ± standard deviation in the different treatments based on daily average micrometeorological data. In 2014, C includes CC, CW, CM, CS and X includes XC, XW, XM, XS (N = 4×365 = 1460), and in 2015, data were obtained from the previously CC and XC sites (N = 2×365 = 730) during the whole year and in August, when all treatments were conducted over the same time period (2014: N = 4×31 = 124; 2015: N = 2×31 = 62). Soil moisture: vol/vol%, temperature (°C)

We observed an increase in soil temperature in the drought-treated plots in both years, which were side effects of precipitation exclusion (Table 1).

#### Substrate-induced respiration

To estimate soil microbial biomass, in 2015, we monitored the metabolic activity of the soil microbial communities on a monthly basis with substrate-induced respiration (SIR) based on the method of Anderson and Domsch [38]. We did not convert the SIR values to soil microbial biomass because of the uncertainty regarding the proper conversion factors used by many studies. Soil samples were taken monthly from May to November, corresponding to the same timeframe as the treatments. Sampling always occurred a few days before the treatments were conducted each month. We took small subsamples of soil from all of the plots using plastic tubes of 12 cm in length and 0.5 cm in diameter. This method allowed us to take soil samples from the upper 10 cm of the soil with a small disturbance effect. From each plot, we took ten subsamples, incorporating as much heterogeneity as possible, and they were mixed. In that way, we obtained approximately 30 g of soil representing the whole plot. The gravimetric water content of the samples was set to 50% of their water holding capacity. Two grams from each sample was measured in 25 cm^3^ vials, and the vials were hermetically covered with butyl rubber plugs and kept in a water bath at 22°C for a 3-day pre-incubation period. The vials were then opened, and the headspace of each was ventilated, ensuring that the starting CO_2_ concentration was the same in all vials. The SIR measurements were carried out by adding a 200 μl 80 mg/cm^3^ glucose solution to each sample, then closing the vials again with the butyl plugs. After 3 h of incubation, the headspace CO_2_ concentrations were determined using the same method as for methane by injection of 250 μl gas samples into a flame ionization gas chromatograph (FISONS GC 8000) along with a methanizer. The SIR rates were calculated as μg CO_2_-C*g soil^-1^*h^-1^. The SIR rates were converted into microbial biomass-C using a conversion factor, 23, obtained from the microbial biomass C estimate obtained for this soil according to the fumigation extraction method [39], i.e., MBC = SIR x 23. The conversion of SIR to MBC is a common practice because good correlation has been found between the two values [40–43].

#### Sampling of microarthropods

For sandy soils, for which traditional soil extraction methods are not appropriate, Liu et al. [29] suggested using pitfall traps, although this method may underestimate euedaphic species. In our experiment, the mesofauna was sampled with EDAPHOLOG probes. In this probe, commercially available horticultural clay granules are used as the medium between the soil and the trapping part, which collects animals from the upper 0–10 cm of the soil based on pitfall trapping and the horizontal movement of soil-living animals. The EDAPHOLOG monitoring system continuously detects microarthropods falling down into the trap with an opto-electronic sensor and records the body sizes and the time of capture [44]. Due to the sandy soil, especially in the drought treatments, sand particles falling into the traps resulted in several miscounts in opto-electronic sensing; therefore, in this study, we used only the biological samples from the traps, i.e., the captured animals stored in 70% ethanol in the plastic tube at the bottom of the trap. Repeated core samplings should be avoided in fine-scaled climatic experiments. EDAPHOLOG, unlike traditional soil extraction methods, is able to sample and monitor mesofauna from sandy soils for a longer time in a non-invasive way. To catch animals with EDAPHOLOG requires microarthropods to actively move into the trap horizontally, as in the case of pitfall traps; therefore, the activity density of the animals (the number of individuals in the traps derived from their abundance and activity) was detected during the experiment. Although the use of this tool was proven to be comparable with traditional methods (Dombos et al., 2017), the capture efficiency of EDAPHOLOG probes in sandy areas was compared with that of traditional sampling methods (soil extraction and pitfall traps) in an additional short field test (see S3 File). Although the capture of animals was low in this field test, the rates of animal AD values did not significantly differ among the different climate conditions, showing that EDAPHOLOG did not affect the response of soil animals to the climatic treatments. In addition, all the AD data were detected with the same type of EDAPHOLOG trap; therefore, all the data are comparable within the experiment.

The probes were placed close to the centre of each plot to prevent the effects of cross-treatment migration. The first year (2014) was considered as the pretreatment year; the traps were inserted into the soil at the beginning of July, and the traps were emptied at the end of November. The traps were monitored throughout this period to prevent errors resulting from failures or clogging of the probes. AD values were obtained for 5 months. In 2015, the traps were emptied every month from April to November following the time schedule of the experimental actions in the treatments; therefore, the AD values refer to one-month-long collections.

Collembola and Oribatida were identified to the species level under a Leica MZ75 microscope according to Bretfeld and Dunger [45], Fjellberg [46], Hopkin [47], Jordana [48], Mahunka [49], Pérez-Iñigo [50], Potapow [51] Stach and Stach [52], Thibaud et al. [53], and Weigmann [54]. In the cases of four specimens of *Folsomia* and two specimens of *Bourletiella*, species-level identification was not possible because of the very young age of the specimens. For further analysis, collembolan species were categorized into three groups, namely, surface-living (epedaphic), vegetation-living, and soil-living (hemiedaphic and euedaphic) species according to the above literature (Table A in S2 File). Mites, except Oribatida, were identified to main groups (Mesostigmata, Prostigmata, Astigmata) [55], and these groups were used for further analysis.

### Data analysis

#### Activity density and species richness of microarthropods

Data obtained from 2014 were corrected by ln(x+1) transformation before performing comparisons of the extreme drought-treated and control sites with Student’s t-tests (Bonferroni’s Type I error correction was made).

In 2015, across the monthly samplings, we had to exclude several samples from the investigation because in some cases, water flooded the samples or spiders inhabited the funnels (mean and SD of data are presented in Tables C and D in S2 File). Because of the missing data and the complexity of the experiment (i.e., the different timeframes of the manipulations), we could not use the statistical design from 2014. To solve this problem, activity density data obtained in 2015 were also analysed after transformation, but we applied two different methods: 1. normalization (relative activity density, RAD) and 2. ordinal scaling (for a detailed description of ordinal scaling, see S1 File).

To make the data of the different orders comparable, we applied the normalized (relative) activity density (RAD). In that case, the AD for a given month and given plot was divided by the total AD detected during the year for the given treatment separately for each mesofaunal group.

To explore the differences in the RAD and SIR data in 2015, we used multivariate ANOVA with two factors: F1 (with or without drought treatment in 2014: X and C) and F2 (C, W, M, and S in 2015). MANOVA was followed by ANOVA with Bonferroni’s Type I error correction for each month to determine the effects of F1 and F2. Note that the levels of F2 dynamically changed according to the starting dates of the treatments in 2015. Statistical analysis was performed with IBM SPSS (V23) software.

For the second statistical approach, which involved the transformation to an ordinal scale, in the case of the separate treatments, we calculated the proportions of the lower AD values before and after the beginning of the treatments in 2015 (i.e., before and after *the first water addition* (W) and at the *beginning of moderate* (M) and *severe* (S) *drought*, respectively). These proportions were compared with Z-tests and Fischer’s exact tests to reveal whether the AD in relation to the control vs. treatment plots significantly changed after the treatment (S1 File contains details about this transformation).

To test whether extreme drought impacted species richness in 2014, we used Student’s t-tests with a Bonferroni correction. We performed a two-way MANOVA with a block design model to test the factor effects on the number of taxa of Collembola and Oribatida in the dataset for 2015. The normality of residuals was checked according to the skewness and kurtosis, while the homogeneity of variance was checked according to the ratio of maximum and minimum variances [56].

## Results

### Effects of the extreme drought pretreatment on activity density and species richness

**Fig 1:**
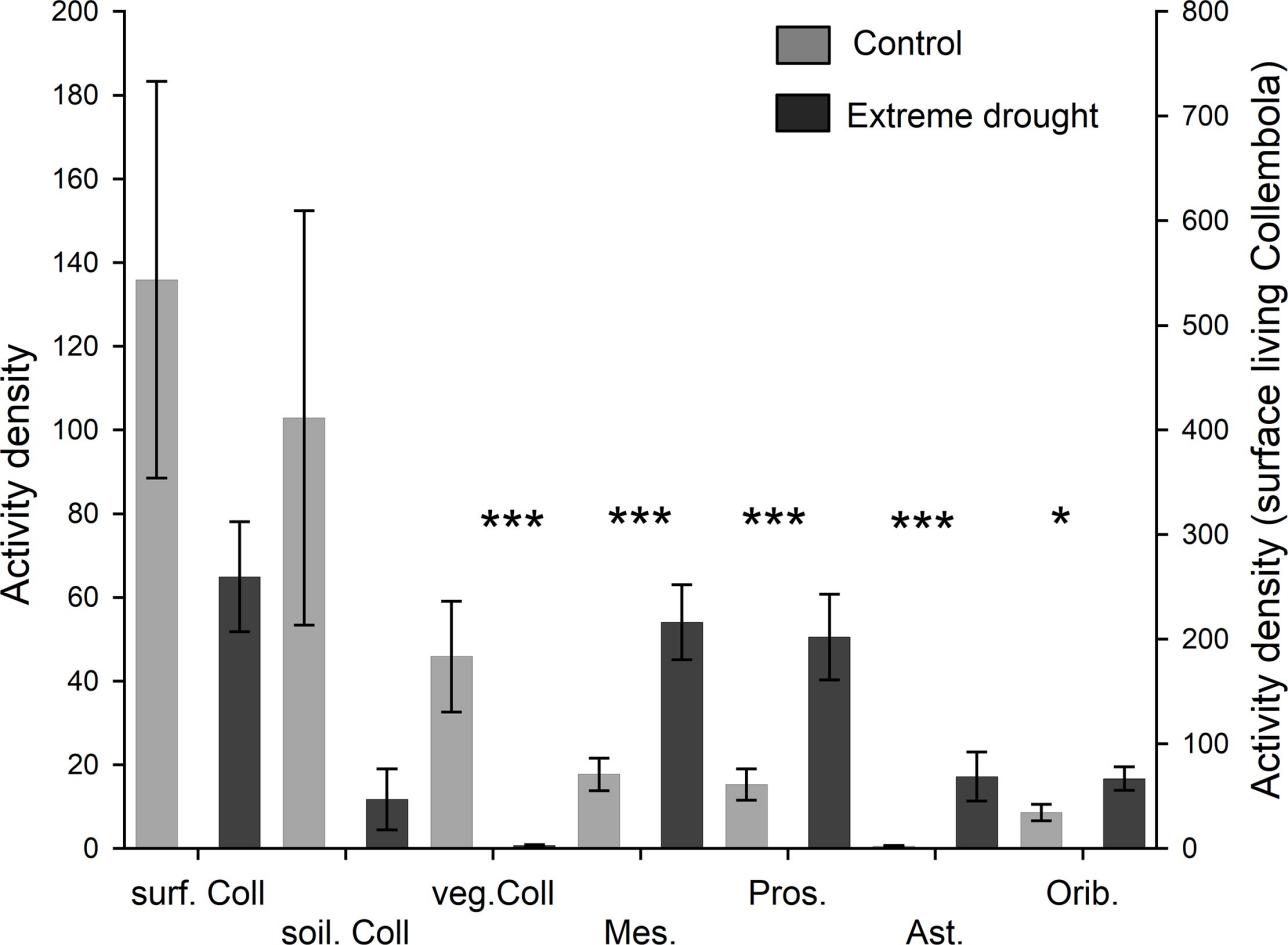
Activity densities of soil mesofauna groups. Mean activity density ± standard error values of different soil mesofauna groups in extreme drought (dark grey) and control plots (light grey) during the experiment. Left axis: soil Coll (hemi- and euedaphic Collembola), veg. Coll (vegetation-living Collembola), Mes. (Mesostigmata), Pros. (Prostigmata), Ast. (Astigmata), Orib. (Oribatida). Right axis: surf. Coll (epedaphic Collembola). Comparisons were performed with ln(x+1)-transformed data according to Student’s t tests (*: p<0.05, ***: p<0.001).

The activity density of all collembolan groups was lower in the year of the extreme drought treatment (2014) in the treated plots than in the control plots. The total number of epedaphic collembolan populations caught in 2014 dropped to 48%, and the activity density of soil-living collembolans also decreased by 90%. However, a significant decrease could have been proven only in the capture of vegetation-living Collembola (Fig 1), for which the decrease was 94%. In contrast, the activity density of all mite groups (Oribatida, Mesostigmata, Prostigmata, Astigmata) significantly increased by 49%, 67%, 70%, and 97% in response to extreme drought (Fig 1).

In the extreme drought treatment, the species richness of Collembola and Oribatida did not change significantly (Table 2). In the case of Collembola, the activity density of common species at the research sites was lower in the extreme drought-treated sites, whereas rare species were present or had a higher AD than in the control plots (Table E in S2 File).

**Table 2:** Species richness of Collembola and Oribatida.

	Treatments (mean ± SD )		
	Control (C)	Extreme drought (X)	p
**Collembola**	3.71±1.27	3.25±1.26	0.645
**Oribatida**	2.04±1.30	3.00±1.57	0.081

Number of taxa of Collembola and Oribatida in extreme drought-treated (N = 24) and control (N = 24) plots in 2014. Comparisons carried out with Student’s t-tests with Bonferroni correction.

#### Effects of the second-year treatments

In contrast to the extreme drought treatment, the less intense drought treatments applied in 2015 (severe and mild) did not show such a clear decreasing pattern. We were not able to prove any significant change in relative activity densities between the different drought treatments compared to the control treatments in any microarthropod group (for analyses, see S1 File). Therefore, despite analysing the activity density data summed across the whole year, we investigated the ordinally scaled differences between the treatments and controls obtained in months before and after the treatment implementation. For this, we constructed the measure of “activity density difference” (ADD, see Methods and S1). We counted the cases when the ADD values of the different mesofaunal groups were relatively lower in the treatment plots (compared to the control). In Table 3, these negative cases are shown according to whether they occurred in monthly captures before or after the beginning of the separate treatments. In the case of epedaphic Collembola, negative effects of both severe and mild drought occurred in relatively high proportions, especially under severe drought (CS-CC), indicating that the populations could not cope with abiotic stress. More frequent negative effects were also found in the plots treated with severe or mild drought and previously treated with extreme drought (XM-XC and XS-SC), although these effects were not statistically significant.

**Table 3:** Comparison of activity density difference (ADD) proportions.

Compared treatment pairs	Percentage of times when the AD values were relevantly lower than those in the control plots (sample sizes)		one-sided Z	Fischer’s exact test p
	before treatment	after treatment		
CM-CC	11% (18)	30% (10)	-1.25 ns	0.32
XM-XC	6% (16)	14% (7)	-0.68 ns	0.51
**CS-CC**	**0 (16)**	**62% (13)**	-3.69 ***	**<0.001**
XS-XC	21% (14)	43% (14)	-1.21 ns	0.42
CW-CC	42% (12)	21% (14)	1.11 ns	0.4
XW-XC	18% (11)	33% (15)	-0.86 ns	0.66

Comparison of activity density difference (ADD) percentages before and after treatment for epedaphic Collembola. Z-test, 2015 data. Percentage data were derived from ordinal scaling. Treatment codes: first character indicates the pretreatment in 2014 (control, C; extreme drought treatment, X), second character indicates the consecutive treatment in 2015 (control (C), water addition (W), moderate drought (M) and severe drought (S).

(*: p<0.05, ***: p<0.001).

In the case of water addition, the results were not as obvious and not significant. We found a lower percent negative difference after treatment in the case of the CW-CC comparison, whereas in the XW-XC comparison, we found the opposite result (Table 3). Other mesofaunal groups showed no significant differences on the basis of this approach.

During the second year, two-way MANOVA did not reveal significant differences in the species richness of Collembola or Oribatida among the previously X- and C-treated plots (F1) or among the C-, W-, M-, and S-treated (F2) plots (Collembola: Wilk’s lambda = 0.857 and 0.809 with p = 0.16 and 0.605, respectively; Oribatida: Wilk’s lambda = 0.819 and 0.792 with p = 0.90 and 0.553, respectively).

The MANOVA of the SIR data showed no significant differences among treatments C, W, M, and S in 2015 (p>0.05) independently regardless of whether they experienced an earlier stress effect in 2014 (X) or not (C) (Table 4).

**Table 4:** Microbial biomass.

Microbial biomass C (mean ± SD)				
pretreatment	treatment in 2015			
	C	W	M	S
X	55.9 ± 24.3	61.2 ± 17.2	48.6 ± 15.5	54.1 ± 10.2
C	45.4 ± 19.0	50.9 ± 12.1	47.1 ± 11.1	52.4 ± 13.0

Mean and SD of microbial biomass C (μg C/g soil; derived from SIR) data from 2015 (7 months and 6 replicates). Rows indicate the previous (2014) treatments: extreme drought (X) and control (C). Columns indicate the treatments in 2015: control (C), water addition (W), moderate (M) and severe drought (S).

#### Legacy effects of extreme drought and the agonistic and antagonistic effects of the treatments

We found a legacy effect of the extreme drought pretreatment only in the case of the SIR data. Significant differences were detected between treatments in the previous year (F1: X and C) with p<0.01; follow-up ANOVA models for the months of May, June, July and August revealed that the SIR in previously X-treated sites was significantly higher (p<0.05); however, for later months, no significant differences were found (p>0.05).

According to MANOVA, we found no agonistic or antagonistic effects of the extreme drought pretreatment or the consecutive treatments (data are shown in S2 File).

#### Description of the soil mesofauna

Overall, at our study site, we found 24 species of Collembola (Table A in S2 File), whereas at the plot level, the mean species number was relatively low (4.13±1.28 (SD)). The total number of Collembola individuals across the two years was 74,400, of which 89.5% belonged to epedaphic and 7% belonged to hemiedaphic-euedaphic species. We found considerably high interannual changes in species composition (see Table A in S2 File). Species found in the area are mainly xerothermophilous species. The epedaphic group was strongly dominated by *Entomobrya nigriventris*, accounting for 99.1% of the epedaphic species. The total number of mite individuals was 12,250 in the two years. Acari was dominated by mesostigmatid (52%) and prostigmatid (23%) mites, but Oribatida (11%) was also present in a considerable number. Oribatida contained 22 species (Table B in S2 File), with a mean species number at the plot level of 1.10±1.27 (SD), and was dominated by xerophilous species such as *Scutovertex sculptus* and *Passalozetes perforatus*. For a further detailed description of species-specific responses to the treatments, see S4 File.

#### Dynamics of mesofauna

**Fig 2:**
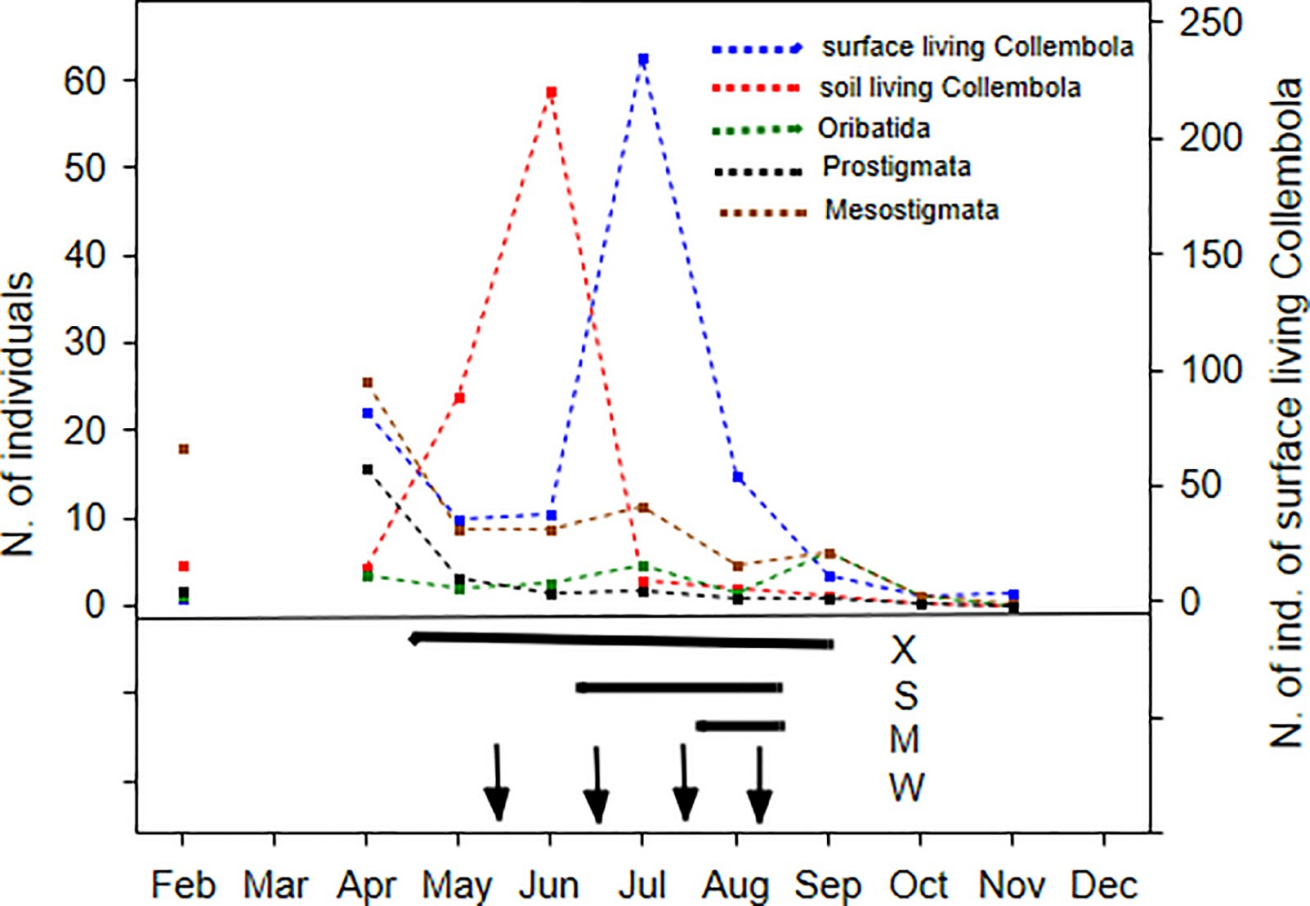
Dynamics of soil mesofauna. Monthly dynamics of the total activity density of different microarthropod groups in the control plots in 2015. Under the figure of these dynamics the timing and duration of the treatments are shown, to see parallel where the treatments possibly affect these natural dynamics. Note: epedaphic Collembola: blue, euedaphic Collembola: red, Oribatida: green, Prostigmata: black and Mesostigmata: brown dotted line. Horizontal bars denote the time periods of the different drought treatments: for treatment 1: X: extreme drought in 2014; for treatment 2: S: severe drought, M: moderate drought in 2015, vertical arrows show water addition events. X axis: time (month) and y axis: number of individuals caught (epedaphic Collembola are on the left y axis).

Fig 2 shows the activity density peaks of the different soil microarthropod groups in the control plots in 2015, revealing that soil mesofauna groups reached their maximum population AD in different time periods during the different seasons. The AD of the epedaphic Collembola populations was high from mid-April to the end of September in the untreated plots, which coincided with the S and partly with the M drought treatments. However, among other microarthropod groups, AD increases occurred only partly during the times that these treatments were implemented. The timing of the extreme drought treatment (X) in the previous year seems to overlap with the active periods of all of the microarthropod groups.

## Discussion

### Immediate effects of the extreme drought pretreatment

One of the most important environmental factors influencing soil mesofaunal behaviour, abundance, and life history is soil moisture [14–19]. Up to 2018, approximately 30% of publications on climate change experiments focused on water regime changes, which resulted in diverse responses. In most field experiments, precipitation reduction induced a negative change in the abundance or density of soil Collembola [17,23,57–62], and adding extra precipitation induced positive responses among soil animals [18,63,64]. In this study, due to the extreme drought treatment (X), the activity density was reduced among all collembolan groups (significant only in the case of vegetation-living Collembola). The observed overall decrease in AD was what we expected and was consistent with other short- and long-term experiments that quantify abundance or density [17,19,62].

Interestingly, unlike Collembola and our expectation, all Acari groups showed an increase in their AD in the extreme-drought plots. Opposite responses of mites and Collembola have been reported in several cases [17], but in many other cases, they responded in the same direction eg. [18,19,63]. Extreme dry conditions or summer drought may have little effect on mite assemblages living in dry ecosystems. Moreover, Liu et al. (2017) suggested that the AD of oribatid mites decreases with increasing soil moisture. This mite group had a higher abundance in a drier grassland than in a wetter grassland [65], and the main Acari groups were also not significantly influenced by summer drought treatments in other grasslands [16,22] or in a dry heathland [30]. Negative effects of drought on oribatid mite assemblages were revealed mainly for closed (woodland) habitats [17,64,66]. The abovementioned results (except Wu et al. [18]) were found in soils with good water retention ability, whereas our study addresses sandy soil, and this condition may have modified the effects of drought on soil fauna in comparison to those found in related studies.

### Effects of mild precipitation changes

Moderate drought is characteristic of the experimental area. In the second-year treatments, although the degree of drought reached the soil moisture content level of the extreme drought (X) pretreatment plots in the previous year, soil-living Collembola and Acari did not show any change in response to moderate or severe drought, contrary to our expectations. We did not find any differences in the species richness of Collembola. Only epedaphic Collembola showed a negative response to the severe drought treatment. The lack of response is common in drought experiments, and the abundance of mesofauna showed little response to the treatments in several cases. Neutral results were found for springtail and mite communities in a fescue field, Mediterranean shrubland and temperate heath, grass and moorland [16,22,23,30]. Even in a poplar shrubland close to our research area, Petersen [23] did not find any changes in collembolan assemblages in drought or warming treatments. This suggests that soil animals can cope well with low levels of environmental fluctuations, especially in areas where natural disturbance is frequent, such as in semiarid sand steppes.

In the water-addition plots, 18.8% of the annual precipitation was added, but the mean monthly soil moisture did not change during the year. Although soil biota require a constant amount of moisture, unlike our expectations, water addition did not increase the microbial biomass or the AD of mesofauna, as in the study of Lindberg and Persson [67]. In several cases, the activity density of invertebrates has been reported not to be influenced by increased precipitation [68]. In the case of soil mesofaunal abundances and even activity densities, further examples exist for the lack of changes in irrigation treatments in arid ecosystems [15,29]. Our study and previous works demonstrate that sporadic extra precipitation in semiarid sandy ecosystems cannot compensate for the effects of drought and is not sufficient to increase microbial biomass or mesofaunal AD. However, precipitation experiments usually have a larger effect over the long term [21], and our experiment may have been too short to detect changes.

### Legacy effects of the extreme drought pretreatment

Although extreme drought had a considerable effect on the soil arthropod assemblages in the pretreatment plots, in the subsequent year, contradictory to our expectations, we did not find any significant effect in AD or richness between the previously treated and control sites. Surprisingly, in the second year, soil moisture was higher at the previously extreme drought-treated sites than at the other sites. This result might have been caused by the mulching effect of dead plant material on the ground and from decreased evapotranspiration because many perennial plants died. This moisture surplus and increased dead material could have caused the higher microbial biomass (SIR) found in the X-treated sites in the second year. Detritus and increased microbial biomass as food resources could have a positive bottom-up effect [18]. We suggest that the higher moisture and resource content of the soil led to a higher activity and number of individuals of Collembola. Mite assemblages may be more affected by actual conditions than by previous climatic changes [16,69]. For each Acari group, the advantage of the drought treatment in terms of causing a higher AD in the first year disappeared in the second year, i.e., drought in the previous year did not apparently influence the activity density values in the second year. In addition, the drought treatment in the second year was not as long as the treatment in the first year; therefore, it should have had lower effects on the Acari groups.

Contrary to our expectations, repeated drought, i.e., severe drought (S), did not amplify the effects of previous extreme drought. However, considering that no similar study has addressed the activity density of microinvertebrates, our findings are consistent with the results of Holmstrup et al. [70]. They found the same patterns in the abundance of enchytraeids.

### Timing and duration are more important than severity of the treatment

In our experiment, the different effects of extreme, moderate and severe drought can be attributed to the duration of the treatments rather than their severities, i.e., the changes in soil moisture contents themselves. The extreme drought treatment was conducted for five months and overlapped the seasonal dynamics and peaks of the AD of all soil mesofauna groups investigated. In contrast, moderate and severe drought events lasted for one and two months, respectively, and overlapped with the peak of epedaphic Collembola but were out of the climate window of other species. Independent of treatment duration, the decrease in soil moisture in the different drought treatments (X, M, and S) similarly reached permanent wilting points in terms of soil moisture content. Even after extra precipitation, the water infiltrated or evaporated rapidly. According to the results from the water addition treatment, we suggest that precipitation quantity is not the only limiting factor in these ecosystems, and the frequency and timing of precipitation events seem to also influence the assemblages of soil mesofauna.

### Additional methodological and ecological aspects

In most studies on soil microarthropods, the usual practice is sampling after or at the end of the treatments only a few times. However, environmental anomalies and extremities may affect different species in different time windows throughout the year. Thus, inappropriate sampling may result in a lack of responses. Soil extraction is only capable of presenting a temporary picture and cannot show changes in soil mesofauna communities continuously. The use of continuous, non-invasive, standardized methods for monitoring soil mesofauna is lacking so far. In our experiment, we used a new sampling method, which involves the continuous monitoring of AD changes among soil mesofauna in sandy soils, where other traditional methods are difficult to implement and not effective. With this non-invasive method, we were able to follow the activity density processes of different microinvertebrate assemblages throughout the whole year, especially in the activity period of arthropods, and this method can be utilized in long-term climate change experiments with minimal disturbance.

Drought influences the soil mesofauna community in two ways: reduced soil moisture acts as a main factor, while higher temperature usually serves as a side effect. In our experiment, different increases in soil temperature occurred during drought treatments; however, we considered them to be negligible. Warming has considerable effects on soil-dwelling biota in northern ecosystems or over the long term [21], whereas it has fewer effects in temperate ecosystems [16,18,19]. In these regions, the side effects of warming (i.e., desiccation) are more important than its direct effects *per se* [16].

Using pitfall-like traps, one cannot sample the entire microarthropod assemblage. Such traps mainly measure the activity densities of species with good locomotory ability [71]. Changes in the activity density of microinvertebrates may be the result of two factors: microarthropods disappearing from the community or remaining in the area but in an inactive state. By using pitfall-like trapping, it is not possible to clearly distinguish the two processes. However, in both cases, their ecological functioning in the community can be evaluated with this method because their ecological functioning (i.e., feeding on resources) is correlated with their activity density. These changes in arid ecosystems should be further investigated.

## Conclusion

Groups of soil mesofauna in fluctuating environments, such as dry sand steppes in central Hungary, are adapted to extreme conditions. Although extreme events can change their activity densities, they seem to be able to cope with changes over a short time. Extreme drought had a greater effect on the activity density of soil mesofauna than severe or moderate drought. However, as the soil moisture in all cases reached the same minimum level, we can state that the timing and duration of a drought event seem to be more important in affecting soil biota than the degree of soil moisture decrease. Because of its low water holding capacity, water does not remain in the sandy soil for a long time, so extra precipitation does not have a positive effect on the soil mesofauna or microbial biomass. The fact that soil moisture and microbial biomass increased at previously disturbed sites may be an example of why climate change factors are reported to have different effects over the short and long term.

## Supporting information

S1 File Data transformations and analysis related to the article. (PDF)

S2 File Figures and tables supporting the results of the article. Fig A Spatial layout of plots at the experimental site Fig B Temporal dynamics of soil moisture in the different treatment plots Table A Collembola species captured at the study site during the experiment Table B Oribatida species captured at the study site during the experiment Table C Mean number of captured Collembola individuals in 2015 across 4 treatment levels Table D Mean number of captured Acari individuals in 2015 across 4 treatment levels Table E Mean (SD) values for the collembolan species in 2014
(PDF)

S3 File Supplementary experiment to evaluate the catchability of EDAPHOLOG probes in sandy soils. (PDF)

S4 File Species-specific responses related to the article. (PDF)

## Abbreviations

AD: activity density
RAD: relative activity density
SM: soil moisture
