# Supplementary material for: Effects of single and repeated drought on soil microarthropods in a semi-arid ecosystem depend more on timing and duration than drought severity
Source: PLoS One. 2019 Jul 18;14(7):e0219975. doi: 10.1371/journal.pone.0219975 (PMC6638988; doi:10.1371/journal.pone.0219975)
Supplement: S1 File — (PDF) [file pone.0219975.s001.pdf]

Supplement 1

Relative activity density (RAD)

In case of normalized (relative) activity densities (RAD), activity density (AD) of the given month and given plot was diveded by the total AD detected during the year at the given treatment, separately for each mesofaunal groups.

$\mathrm{RAD_{tmb}} = \mathrm{AD_{tmb}} / \sum_{\mathrm{m=4}}^{11} \overline{\mathrm{AD_{tm\cdot}}}$, where the *t* is for treatment factor combinations (CC, XC, CW, XW, CM, XM, CS, XS), *m* is for time expressed in month (m=4, 5, …, 11), *b* is for blocks (b=1, 2, …, 6), $\overline{\mathrm{AD_{tm\cdot}}}$ is the mean activity density in month *m* treated by *t* calculated over all blocks *b*.

In 2015 MANOVA has not found significant difference among the relative activity density values (RAD) either of different treatment levels of F1 (the effect of extreme drought from the pre-treatment) or the ones of F2 (the effects of repeated drought events) and the interactions of the two factors in any case of microarthropod groups (p>0.05; data are shown in S1 Table A).

Relative activity densities (mean± SD) in different treatment combinations					
Mesofauna groups	2014	**C**	**W**	**M**	**S**
surface living Collembola	**X**	0.11 ± 0.19	0.12 ± 0.18	0.14 ± 0.26	0.12 ± 0.22
	**C**	0.1 ± 0.14	0.1 ± 0.2	0.12 ± 0.18	0.13 ± 0.17
soil living Collembola	**X**	0.12 ± 0.44	0.11 ± 0.46	0.14 ± 0.25	0.13 ± 0.32
	**C**	0.13 ± 0.51	0.14 ± 0.46	0.13 ± 0.47	0.13 ± 0.43
vegetation living Collembola	**X**	0.13 ± 0.38	0.13 ± 0.3	0.14 ± 0.43	0.12 ± 0.28
	**C**	0.12 ± 0.24	0.14 ± 0.36	0.13 ± 0.31	0.12 ± 0.31
Mesostigmata	**X**	0.11 ± 0.19	0.11 ± 0.2	0.11 ± 0.2	0.12 ± 0.25
	**C**	0.11 ± 0.21	0.12 ± 0.31	0.12 ± 0.21	0.12 ± 0.17
Prostigmata	**X**	0.12 ± 0.27	0.12 ± 0.27	0.12 ± 0.23	0.12 ± 0.24
	**C**	0.11 ± 0.27	0.12 ± 0.28	0.12 ± 0.21	0.12 ± 0.21
Oribatida	**X**	0.11 ± 0.21	0.11 ± 0.18	0.1 ± 0.13	0.12 ± 0.18
	**C**	0.09 ± 0.16	0.11 ± 0.21	0.12 ± 0.15	0.12 ± 0.13

S1 Table A. Relative activity density data (mean± SD) from 2015. Rows indicate the previous (2014) treatments: extreme drought (X) and control (C). Columns indicate the treatments in 2015: control (C), water addition (W), moderate (M) and severe drought (S).

Activity density difference (ADD)

Activity density difference (direction and magnitude, ADD*):* Species from soil mesofauna, assumed they are present, usually are able to establish an extremely large population size within a short time. To express the *direction* and the *magnitude of the relevant difference* between two values, first we converted the activity density data to ordinal scale defining activity density *rate* categories as presented in S1 Table B.

ACTIVITY DENSITY	RATE CATEGORY FOR SURFACE LIVING COLLEMBOLA AND ACARI	
0-10	*I. (low)*	i.e. the appearance of the species is almost random
11-100	*II. (medium)*	i.e. the species are present, but their activity density is moderate
above 100	*III. (high)*	i.e. the species are abundant
ACTIVITY DENSITY	RATE CATEGORY FOR SOIL AND VEGETATION LIVING COLLEMBOLA	
0-10	*I. (low)*	i.e. the appearance of the species is almost random
11-50	*II. (medium)*	i.e. the species are present, but their activity density is moderate
above 50	*III. (high)*	i.e. the species are abundant

S1 Table B. Activity density (number of individuals found in a trap in a trapping period) rate categories, calculated from their values. We introduced two different rate categories because of the different activity density values of the two groups. Surface living Collembola and Acari were found in higher number in the traps. To obtain differences in their activity density, higher values had to be used for their categories than for other Collembola groups.

From the ordinal data, we defined the ADD (as their direction and magnitude) while comparing the activity density values of factor combinations CW, CM, CS (control in 2014, treated in 2015) to CC (control in both years), and XW, XM, XS (treated in both years) to XC (treated in 2014, control in 2015) in each block.

The *difference* is considered (see S1 Fig A) as

- ±1 if the two compared activity density values are from rate categories

  * I and II, assumed that their absolute difference is greater than 10 or

  * II and III, assumed that their difference is greater than 50 or

  * II and II (both), assumed that the greater value is at least twice as high as the lower one
- ±2 if the two compared activity density values are from rate categories I and III
- 0 else.

The values are negative if the control value (CC or XC) is higher, that is to say, activity density is supposed to be relevantly higher in control samples.

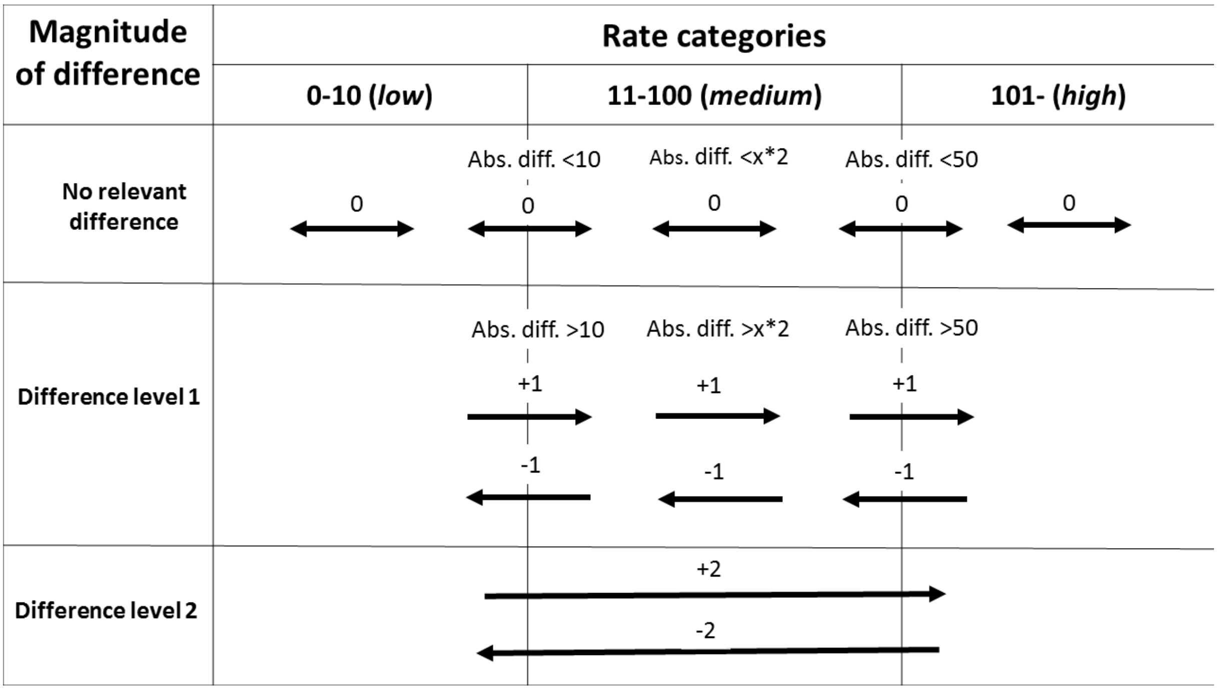

S1 Fig A. Calculation of activity density differences (direction and magnitude) comparing treatment and control factor-pair values of year 2015. Criterions of differences are presented above the arrows. *Magnitude of difference*: “no relevant difference” (0); “difference level 1” (±1), “difference level 2” (±2). “x” means the lower value from the two compared activity density values.
