# Supplementary material for: Effects of single and repeated drought on soil microarthropods in a semi-arid ecosystem depend more on timing and duration than drought severity
Source: PLoS One. 2019 Jul 18;14(7):e0219975. doi: 10.1371/journal.pone.0219975 (PMC6638988; doi:10.1371/journal.pone.0219975)
Supplement: S2 File — Fig A Spatial layout of plots at the experimental site Fig B Temporal dynamics of soil moisture in the different treatment plots Table A Collembola species captured at the study site during the experiment Table B Oribatida species captured at the study site during the experiment Table C Mean number of captured Collembola individuals in 2015 across 4 treatment levels Table D Mean number of captured Acari individuals in 2015 across 4 treatment levels Table E Mean (SD) values for the collembolan species in 2014 (PDF) [file pone.0219975.s002.pdf]

Supplement 2

S2 Fig A. Spatial layout of plots at the experimental site. X indicates the sites treated with extreme drought (factor 1), plots without X were used as controls in the first year (2014). In the consecutive year (2015) mild precipitation changes (factor 2) were used with 4 four levels: water addition (W, blue plots), control (C, green plots), moderate drought (M, light yellow plots) and severe drought (S, dark yellow plots). The study site is surrounded by a fence (dashed red line).

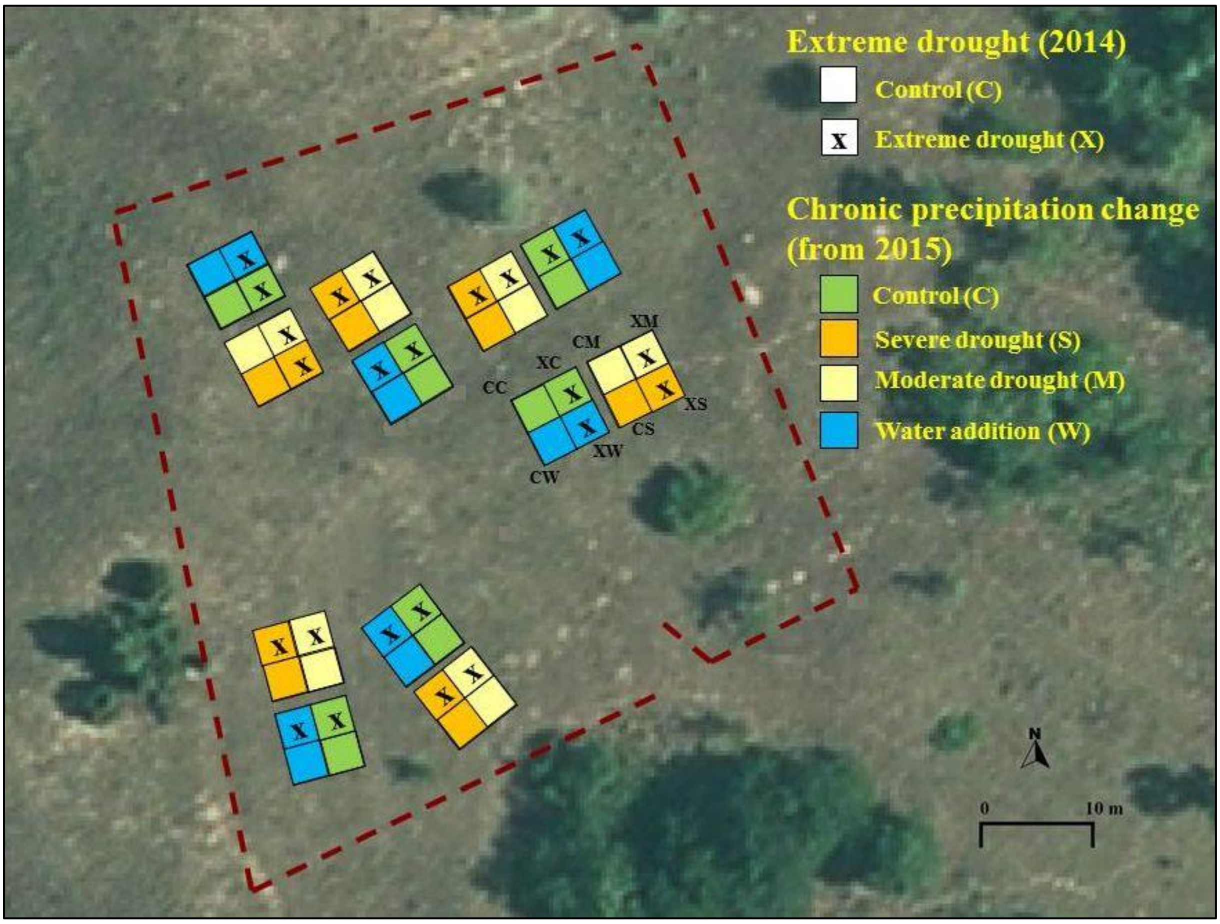

S2 Fig B. Temporal dynamics of soil moisture in the different treatment plots. y axis: Soil moisture (SM) (vol/vol%), x axis: time (days). A: first year of treatment (2014), B: second year of treatment (2015) showing the difference between previously X treated and untreated sites, C: second year of treatment (2015) showing the difference between the four levels of treatments. Red dotted line: SM at extreme drought treated sites, green dotted lines: SM at control sites, blue dotted line: SM at water addition sites, orange dotted line: SM at moderate drought treated sites, brown dotted line: SM at severe drought treated sites. W indicates the time when water addition treatments were applied. M, S, X represent the time frame of different treatments, respectively: moderate drought, severe drought, extreme drought).

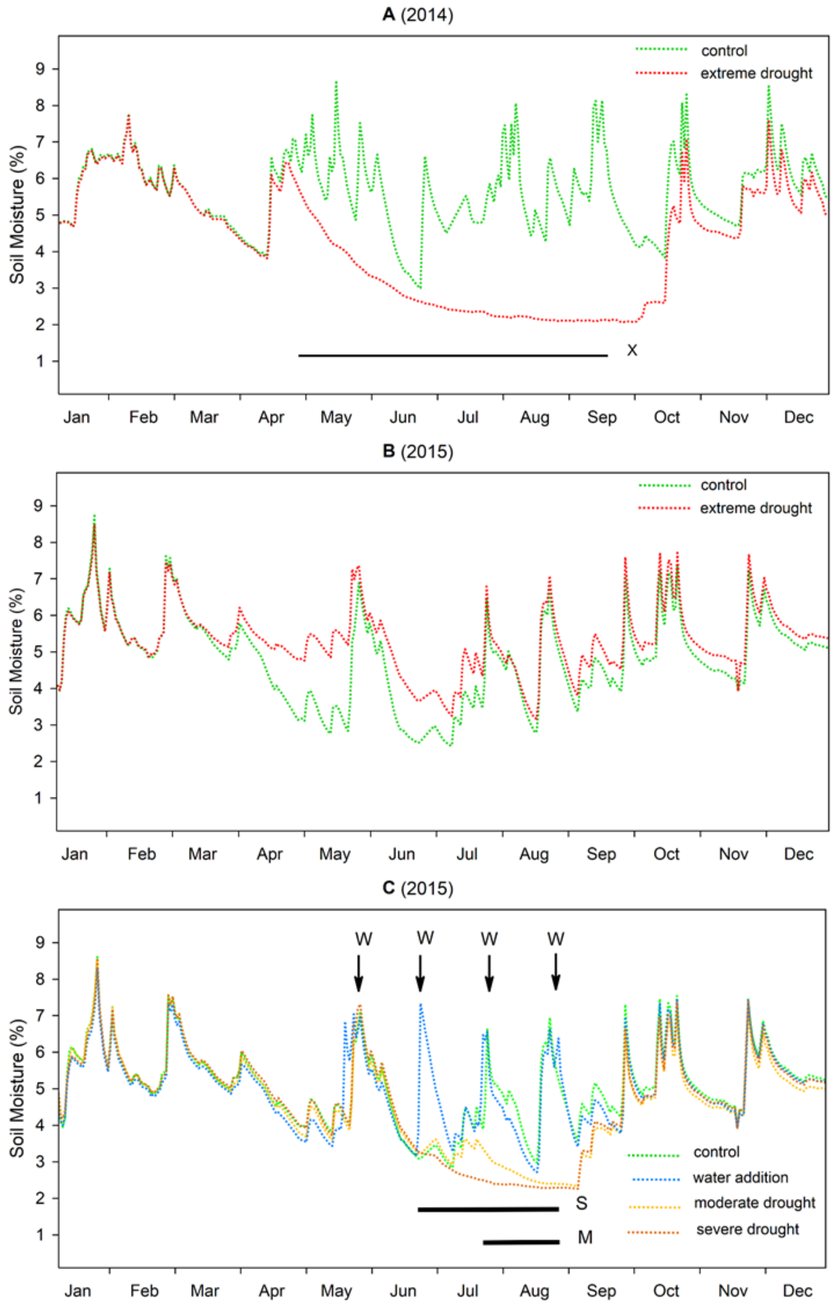

S2 Table A. Collembola species captured at the study site during the experiment.

Collembola species	2015 Apr.-Nov.	2015 Jul.-Nov.	2014 Jul.-Nov.	main habitat	temperature and moisture	vertical stratification	category
**Most frequent species**	number of individuals						
Arrhopalites caecus (Tullberg, 1871)	574	93	381	synanthropic, caves [1]	troglophil, mesophil [2, 3]	hemiedaphic to euedaphic [2, 4, 5, 6]	soil living
Entomobrya nigriventris Stach,1930	46803	19023	19271	grassland [7]	xerothermophil [8]	epedaphic	surface living

Lepidocyrtus cyaneus Tullberg, 1871	488	329	2	open ecosystem [1, 9]	mesophil [10]	epedaphic [5, 11]	surface living
Proisotoma minuta (Tullberg, 1871)	556	273	2286	compost, ruderal [1]	thermophil, hygrophil [8, 10, 12, 13]	hemiedaphic [5]	soil living
Sphaeridia pumilis (Krausbauer, 1898)	281	206	1103	open ecosystem [1]	mesophil to hygrotolerant [3, 8, 12, 14]	vegetation living, hemiedaphic [2, 12, 14]	vegetation living
Xenylla maritima Tullberg, 1869	2372	402	10	open, arid ecosystem [1]	xerophil [8, 15]	hemiedaphic [16]	soil living
**Rare species**							
Anurophorus pseudolaricis Loksa, 1978	3	0	0	moist habitat [17]	hygrophil [17]	hemiedaphic [17]	soil living
Bourletiellida	1	1	0	?	?	?	not included
Brachystomella curvula Gisin, 1948	7	3	30	steppe ecosystem, ravine forest [1]	xerothermophil [6, 8, 18]	hemiedaphic [6]	soil living
Ceratophysella succinea (Gisin, 1949)	2	0	1	open ecosystem, anthropogenic origin [1]	mesophilous [18]	hemiedaphic [6]	soil living
Cyphoderus albinus Nicolet, 1842	34	6	23	myrmecophil, forest, steppe, flood meadow [1]	thermophil, mesophil to xerophil [6, 9, 11]	euedaphic [6]	soil living
Entomobrya multifasciata (Tullberg, 1871)	7	1	8	open ecosystem, forest [1]	xerothermophilic [8, 15]	hemiedaphic [18]	soil living
Folsomia candida (Willem, 1902)	7	0	5	ruderal, troglophilic [1]	probably thermophil, mesophil [6]	euedaphic [6, 11]	soil living
Heteromurus nitidus (Templeton, 1835)	9	0	1	synanthropic, forests and meadows [1]	hygrophil to mesophil [6, 19]	hemiedaphic to euedaphic [4, 5, 6, 8]	soil living
Orchesella taurica (Stach, 1960)	49	0	2	forest, meadow steppe ecosystem [1] ***new to the Hungarian fauna***	xerophil [1]	hemiedaphic [1]	soil living
Protaphorura serbica (Loksa & Bogojevic, 1967)	0	0	3	open, arid ecosystem [1]	xerothermophil [8, 20]	eudepahic [9]	soil living
Pseudosinella sexoculata Schött, 1902	38	12	0	open ecosystem [1]	?	hemiedaphic [18]	soil living
Sminthurus viridis (Linnaeus, 1758)	18	1	14	open ecosystem, anthropogenic [1]	hygrophil, mesophil [15, 18]	vegetation living [15]	vegetation living
Sminthurus mulitpunctatus Schäffer, 1896	0	0	1	low vegetation of dry habitats [2]	xerothermophil [2, 8, 15]	vegetation living [2, 15]	vegetation living
Sminthurus nigromaculatus Tullberg, 1871	1	0	0	open ecosystems; steppe	xerothermophil [2]	vegetation living [2]	vegetation living

				ecosystem, ravine forests [1]			
Sminthurus maculatus Tömösváry, 1883	0	0	1	steppe [1]	xerothermophil [2]	vegetation living [2]	vegetation living
Willowsia buski (Lubbock,1869)	6	2	2	synanthropic, corticophil, saxicolous and xerothermic grassland [1, 8, 15]	xerothermophil [8, 15]	hemiedaphic [15]	soil living

References:

1 Kaprus I, Shrubovych J, Tarashchuk M (2006) Catalogue of the Collembola and Protura of Ukraine. Praci Derzhavnoho pryrodoznavchoho muzeju NAN Ukrainy 59: 1-164.

2 Bretfeld G, Dunger W (1999) Synopses on Palaearctic Collembola: Symphypleona. Staatlicjes Museum für Naturkunde Görlitz.

3 Russell DJ, Griegel A (2006) Influence of variable inundation regimes on soil Collembola. Pedobiologia 50: 165-175.

4 Gutiérrez-López M., Salmon S, Trigo D (2011) Movement response of Collembola to the excreta of two earthworm species: Importance of ammonium content and nitrogen forms. Soil Biology and Biochemistry 43, 55-62.

5 Henneron L, Aubert M, Archaux F, Bureau F, Dumas Y, Ningre F, et al. (2017) Forest plant community as a driver of soil biodiversity: experimental evidence from collembolan assemblages through large-scale and long-term removal of oak canopy trees *Quercus petraea*. Oikos 126: 420-434.

6 Stierhof T (2003) Collembolengemeinschaften in baden-württembergischen Waldböden. Universitätsbibliothek Giessen.

7 Traser G, Szűcs P, Winkler D (2006) Collembola diversity of moss habitats in the Sopron Region, NW-Hungary. Acta Silvatica et Lignaria Hungarica 2: 69-80.

8 Dányi L, Traser G (2008) An annotated checklist of the springtail fauna of Hungary (Hexapoda: Collembola). Opuscula Zoologica 38: 3-82.

9 Loksa I (1966) Die bodenzoozönologischen Verhältnisse der Flaumeichen-Buschwälder Südostmitteleuropas. Akadémiai Kiadó.

10 Strebel O, Altner H (1961) Weitere Beitrage zur Apterygotenfauna des Siebengebirges und der Rudderberges. Decheniana Beih. 9: 79-106.

11 Hopkin SP (2007) A key to the Collembola (springtails) of Britain and Ireland. FSC publications.

12 Gisin H (1943) Ökologie und Lebensgemeinschaften der Collembolen im schweizerischen Exkursionsgebiet Basels: Inauguraldissertation... vorgelegt der philosophisch-naturwissenschaftlichen Fakultät der Universität Basel von Hermann Gisin. A. Kundig.

13 Potapow M (2001) Synopses on Palaearctic Collembola: Isotomidae. Staatliches Museum für Naturkunde.

14 Pong JF (1993) Biocenoses of Collembola in atlantic temperate grass-woodland ecosystems. Pedobiologia 37: 223-244.

15 Szeptycki A (1967) Fauna of the springtails (Collembola) of the Ojców National Park in Poland. Zakład Zoologii Systematycznej Polskiej Akademii Nauk.

16 Thibaud JM, Schulz HJ, Assalino MMZG, Dunger W (2004) Synopses on Palaearctic Collembola: Hypogastruridae. Staatliches Museum für Naturkunde Görlitz.

17 Loksa I (1978) Mikrohabitate und ihre Bedeutung für die Verteilung der Collembolengemeinschaften in einem Hainbuchen-Eichenbestand. Opuscula Zoologica Budapest 15: 93-117.

18 Buşmachiu G (2011) Collembola (Hexapoda) from the riparian habitats of the Dniester River. Muzeul Olteniei, Craiova. Studii şi comunicări. Ştiinţele Naturii 27: 63-70.

19 Bockemühl J (1956) Die Apterygoten des Spitzberges bei Tübingen: Eine faunist.-ökol. Unters.(Mit 17 Abb. im Text).

20 Pomorski RJ (1998) Onychiuridae of Poland (Collembola: Onychiuridae). Polskie Towarzystwo Taksonomiczne.

S2 Table B. Oribatida species captured at the study site during the experiment.

Oribatida species	2015 Apr.-Nov.	2015 Jul.-Nov.	2014 Jul.-Nov.	main habitat	moisture	feeding
**Most frequent species**						
Scutovertex sculptus Michael, 1879	315	231	228	moss, canopy, grassland [1, 2]	halophil, xerophil [1, 2]	microphytophag [3]
Passalozetes perforatus (Berlese, 1910)	327	177	253	grassland [2]	xerophil [2]	microphytophag [4]
Oribatula divida Mahunka, 1987	193	70	41	grassland (endemic?) [5]	?	?
Latovertex hungaricus Mahunka, 1987	44	15	39	grassland (endemic?) [5]	?	?
Trichoribates trimaculatus (Koch, 1836)	13	4	15	moss, canopy, grassland [2]	xerophil [2]	panphytophag [6]
Zygoribatula glabra (Michael, 1890)	16	3	2	grassland, ruderal [1,7]	xerophil [7]	?
**Rare species**						
Belba sculpta Mihelčič, 1957	2	2	0	forest [8] ***new to the Hungarian fauna***	?	?
Scheloribates fimbriatus Thor, 1930	2	2	4	grassland [5]	?	?
Micreremus brevipes (Michael, 1888)	4	2	1	canopy, lichen, moss [2]	xerophil [2]	microphytophag [6]
Galumna bimorpha Mahunka, 1987	1	1	0	grassland (endemic?) [5]	?	?
Phauloppia lucorum (Koch, 1841)	3	0	0	canopy, lichen [2]	xerophil [2]	panphytophag [9]
Gymnodamaeus bicostatus (Koch, 1835)	1	0	1	forest, canopy, lichen, moss [2]	xerophil [2]	microphytophag [10]
Pilogalumna crassiclava (Berlese, 1914)	1	0	1	forest, ruderal [1,2]	xerophil [2]	?
Tectocepheus sarekensis Trägardh, 1910	1	0	1	euryoecious 2	-	panphytophag [11]
Aleurodamaeus setosus (Berlese, 1883)	1	0	0	forest, moss [5,12]	xerophil [5]	?
Scheloribates pallidulus (Koch, 1841)	2	0	0	forest [2]	hygrophil [2]	panphytophag [3]
Oppiella uliginosa (Willmann, 1919)	1	0	0	forest, moss, canopy [2]	?	?
Scheloribates latipes (Koch, 1844)	1	0	0	forest, grassland [1]	?	microphytophag [13]
Camisia segnis (Hermann, 1804)	2	0	0	canopy, moss [1,2]	xerophil [2]	panphytophag [14]
Zygoribatula exilis (Nicolet, 1855)	0	0	2	canopy, moss [2]	xerophil [2]	microphytophag [15]
Ramusella insculpta (Paoli, 1908)	0	0	1	forest, grassland [1]	xerophil [1]	microphytophag [15]
Ramusella clavipectinata (Michael, 1885)	0	0	1	grassland, moorland [1]	hygrophil [1]	microphytophag [9]

References

1 Weigmann G (2006) Hornmilben (Oribatida). Die Tierwelt Deutschlands, begründet 1925 von Friedrich Dahl. 76. Teil. Goecke & Evers, Keltern.

2 Schatz H (2008) Hornmilben (Acari: Oribatida) im Naturpark Schlern-Rosengarten:(Südtirol, Italien). na.

3 Honciuc V (1993) Taxonomical and zoogeographical researches of oribatid fauna (Acari-Oribatida) in different ecosystems of Danube Delta. Rev. Roum. Biol., Biol. Anim 38: 21-29.

4 Schuster R (1960) Über die Ökologie und Verbreitung von Bodenmilben (Oribatei) am Alpen-Ostrand, insbesondere in der Steiermark. Mitt. naturwiss. Ver. Steiermark 90: 132-149.

5 Mahunka S (1987) A survey of the oribatids of the Kiskunság National Park (Acari: Oribatida). The fauna of the Kiskunság National Park 2: 346-397.

6 Borcard D (1995) Les Oribates des tourbières du Jura suisse (Acari, Oribatei). Faunistique VII. Oribatuloidea (Haplozetidae), Ceratozetoidea. Mitteilungen-Schweizerische Entomologische Gesellschaft 68: 363-372.

7 Höpperger M, Schatz H (2013) Hornmilben (Acari, Oribatida) von Castelfeder (Südtirol, Italien). Gredleriana 13: 71-98.

8 Murvanidze M, Mumladze L (2016) Annotated checklist of Georgian oribatid mites. Zootaxa 4089: 1-81.

9 Siepel H, de Ruiter-Dijkman E (1993) Feeding guilds of oribatid mites based on their carbohydrase activities. Soil Biology and Biochemistry 25: 1491-1497.

10 Skubała P, Kafel A (2004) Oribatid mite communities and metal bioaccumulation in oribatid species (Acari, Oribatida) along the heavy metal gradient in forest ecosystems. Environmental pollution 132: 51-60.

11 Schatz H (1993) Milbenzönosen (Acari) in der Umgebung der Montanwerke Brixlegg (Tirol, Österreich). Verhandlungen-Gesellschaft Für Ökologie 23: 113-113.

12 Paschoal A, Johnston D (1985) Aleurodamaeidae (Acari: Oribatei), a new family of oribatid mites, with a description of *Aleurodamaeus hungaricus*, sp. n. Revista Brasileira de Biologia 29: 21-26.

13 Borcard D (1994) Les Oribates des tourbiéres du Jura suisse (Acari, Oribatei). Faunistique VI. Oppioidea (Thyrisomidae), Hydrozetoidea, Cymbaeremoidea, Oribatuloidea (part.). Mitteilungen der Schweizerischen entomologischen Gesellschaft 67: 363-372.

14 Schatz H (1996) Hommilben (Acari, Oribatida) in Trockenrasenböden des Virgentales (Osttirol, Österreich, Zentralalpen). Wiss Mitt Nationalpark Hohe Tauern 2: 97-114.

15 Olszanowski Z, Niedbala W, (2000) Moss mites (ACARI: ORlBATIDA) from the Slonsk Nature Rereve: Geographic Elements and the types of phagism. Biological Bulletin of Poznan 37: 299-302.

S2 Table C. Mean number of captured Collembola individuals in 2015 across 4 treatment levels (control, water addition, moderate or severe drought). First table (Collembola) includes all of the Collembolan specimens, whereas thereafter separate groups are shown. The rows contain the months and the previous treatments (C: control, X: extreme drought).

		**Collembola (all groups)** (mean ± SD (number of samples))			
**month**	**first treatment**	**Control**	**Water**	**Moderate d.**	**Severe d.**
4	c	244.8 ± 140.7 (6)	307.3 ± 256.1 (6)	648 ± 301.6 (6)	507.2 ± 272.4 (6)
	x	167.8 ± 78.1 (6)	345.8 ± 315.6 (6)	437.8 ± 386.1 (6)	283.5 ± 222.7 (6)
5	c	127.7 ± 96.5 (6)	140.5 ± 149.3 (6)	281.7 ± 198.2 (6)	208.8 ± 82.3 (6)
	x	83.2 ± 62.2 (5)	164.8 ± 157 (6)	137.7 ± 184.7 (6)	98 ± 82 (6)
6	c	168 ± 214.4 (5)	148.8 ± 267.7 (6)	194 ± 231.4 (6)	184.2 ± 161.7 (5)
	x	79.3 ± 94.9 (4)	110.8 ± 182.8 (6)	42.4 ± 37.9 (5)	140.6 ± 193.1 (5)
7	c	347.5 ± 169 (2)	576.3 ± 712.3 (3)	667.5 ± 650.1 (4)	246.8 ± 220.9 (5)
	x	315.5 ± 351.7 (4)	544.3 ± 643.6 (4)	78.3 ± 40.9 (4)	400 ± 421 (5)
8	c	137 ± 190 (5)	307.3 ± 539.2 (4)	141.8 ± 234.2 (5)	18.7 ± 14.8 (6)
	x	111.3 ± 122.5 (6)	213.3 ± 189.7 (4)	50.7 ± 66.3 (3)	20.3 ± 13.8 (6)
9	c	56.8 ± 61.1 (6)	50 ± 55 (6)	57.5 ± 62.2 (6)	32.5 ± 28.8 (6)
	x	80.2 ± 102.9 (5)	125.3 ± 121.1 (4)	21.8 ± 19 (5)	21 ± 16.3 (6)
10	c	6.8 ± 5 (6)	26.3 ± 28 (6)	32.2 ± 30.9 (6)	44.6 ± 25.5 (5)
	x	6.8 ± 8.7 (6)	15.2 ± 13.6 (6)	36.7 ± 32 (6)	20.5 ± 10.7 (6)
11	c	5.3 ± 5.2 (6)	6.2 ± 5.8 (6)	5 ± 1.2 (5)	9.8 ± 5.9 (6)
	x	3.3 ± 3.6 (6)	5.5 ± 5.9 (6)	14.2 ± 7.1 (6)	7.2 ± 6.6 (6)

		**Soil living Collembola** (mean ± SD (number of samples))			
**month**	**first treatment**	**Control**	**Water**	**Moderate d.**	**Severe d.**
4	c	10.3 ± 18.5 (6)	44.2 ± 97.9 (6)	22.5 ± 45.9 (6)	14.7 ± 14.5 (6)
	x	14.7 ± 33 (6)	6.5 ± 9.3 (6)	2.7 ± 2.4 (6)	3.2 ± 3.5 (6)
5	c	42.2 ± 97.4 (6)	13.8 ± 20.8 (6)	27.8 ± 58 (6)	23.7 ± 50.3 (6)
	x	38.4 ± 79.3 (5)	4.2 ± 5 (6)	1 ± 1.7 (6)	3.5 ± 4.8 (6)
6	c	99.4 ± 221.1 (5)	13.3 ± 27.8 (6)	75 ± 164.7 (6)	1 ± 1 (5)
	x	8 ± 14.7 (4)	13 ± 29.9 (6)	0.4 ± 0.5 (5)	1.4 ± 1.5 (5)
7	c	4 ± 4.2 (2)	2.7 ± 2.3 (3)	7 ± 13.3 (4)	0.4 ± 0.5 (5)
	x	2.5 ± 5 (4)	91.8 ± 178.9 (4)	0.3 ± 0.5 (4)	0.8 ± 0.8 (5)

8	c	2.4 ± 5.4 (5)	1.3 ± 1.3 (4)	0.2 ± 0.4 (5)	0 ± 0 (6)
	x	1.8 ± 1.8 (6)	2.3 ± 3.9 (4)	0 ± 0 (3)	6.7 ± 16.3 (6)
9	c	1.5 ± 1.6 (6)	1.2 ± 1 (6)	3.3 ± 6.8 (6)	2.2 ± 4.8 (6)
	x	1.2 ± 1.3 (5)	1.3 ± 1.9 (4)	3.2 ± 3.6 (5)	1 ± 0.6 (6)
10	c	2.2 ± 4.8 (6)	3.8 ± 3.5 (6)	2.2 ± 4.4 (6)	1.4 ± 1.5 (5)
	x	0.2 ± 0.4 (6)	3.2 ± 6.8 (6)	4.8 ± 8.2 (6)	1.3 ± 1 (6)
11	c	1.3 ± 3.3 (6)	3.2 ± 4.2 (6)	2.2 ± 3 (5)	2.5 ± 2.4 (6)
	x	0.5 ± 0.8 (6)	0.2 ± 0.4 (6)	4 ± 4.6 (6)	1.7 ± 3.1 (6)

		**Surface living Collembola** (mean ± SD (number of samples))			
**month**	**first treatment**	**Control**	**Water**	**Moderate d.**	**Severe d.**
4	c	234 ± 144.5 (6)	263.2 ± 279.6 (6)	625.2 ± 313.2 (6)	492.5 ± 278 (6)
	x	153 ± 94.6 (6)	337.5 ± 305.3 (6)	434.7 ± 385.1 (6)	279.8 ± 223.8 (6)
5	c	85 ± 45.5 (6)	126.3 ± 158.7 (6)	253 ± 211.4 (6)	184.8 ± 90.9 (6)
	x	44.8 ± 30.6 (5)	160.2 ± 152.9 (6)	136.5 ± 184.8 (6)	94.2 ± 84.1 (6)
6	c	68.2 ± 60.3 (5)	134.5 ± 272.8 (6)	117.5 ± 191.8 (6)	181.4 ± 161.7 (5)
	x	71.3 ± 98.7 (4)	96.7 ± 150.5 (6)	41.8 ± 37.4 (5)	137 ± 189.3 (5)
7	c	343 ± 164 (2)	573.7 ± 711 (3)	660 ± 636.5 (4)	246.4 ± 221.2 (5)
	x	313 ± 354.4 (4)	451.8 ± 503.4 (4)	77.8 ± 40.1 (4)	399.2 ± 421.6 (5)
8	c	133.8 ± 189.4 (5)	306 ± 538 (4)	141.6 ± 233.7 (5)	18.7 ± 14.8 (6)
	x	109.3 ± 123.7 (6)	210.8 ± 187.4 (4)	50.7 ± 66.3 (3)	13.7 ± 9.7 (6)
9	c	55 ± 60 (6)	48.2 ± 55.5 (6)	53.7 ± 63.9 (6)	26.3 ± 24.6 (6)
	x	78 ± 102.9 (5)	123.5 ± 119.7 (4)	18.2 ± 16.8 (5)	19.8 ± 15.6 (6)
10	c	4.3 ± 2.9 (6)	20.2 ± 30.6 (6)	29.7 ± 29.3 (6)	35 ± 29.6 (5)
	x	6.3 ± 7.7 (6)	10.8 ± 7.4 (6)	28.3 ± 23.4 (6)	16.8 ± 10.5 (6)
11	c	3.3 ± 4.6 (6)	2.8 ± 3 (6)	2.8 ± 2.2 (5)	5.5 ± 4.1 (6)
	x	2.3 ± 3.4 (6)	5 ± 5.7 (6)	8 ± 6.2 (6)	3 ± 3.6 (6)

		**Vegetation living Collembola** (mean ± SD (number of samples))			
**month**	**first treatment**	**Control**	**Water**	**Moderate d.**	**Severe d.**
4	c	0.5 ± 0.8 (6)	0 ± 0 (6)	0.3 ± 0.5 (6)	0 ± 0 (6)
	x	0.2 ± 0.4 (6)	1.8 ± 2.9 (6)	0.5 ± 1.2 (6)	0.5 ± 0.8 (6)
5	c	0.5 ± 0.8 (6)	0.3 ± 0.5 (6)	0.8 ± 2 (6)	0.3 ± 0.8 (6)
	x	0 ± 0 (5)	0.5 ± 1.2 (6)	0.2 ± 0.4 (6)	0.3 ± 0.8 (6)
6	c	0.4 ± 0.5 (5)	1 ± 2 (6)	1.5 ± 2.1 (6)	1.8 ± 2.2 (5)
	x	0 ± 0 (4)	1.2 ± 2.9 (6)	0.2 ± 0.4 (5)	2.2 ± 4.9 (5)
7	c	0.5 ± 0.7 (2)	0 ± 0 (3)	0.5 ± 1 (4)	0 ± 0 (5)
	x	0 ± 0 (4)	0.8 ± 1.5 (4)	0.3 ± 0.5 (4)	0 ± 0 (5)

8	c	0.8 ± 1.8 (5)	0 ± 0 (4)	0 ± 0 (5)	0 ± 0 (6)
	x	0.2 ± 0.4 (6)	0.3 ± 0.5 (4)	0 ± 0 (3)	0 ± 0 (6)
9	c	0.3 ± 0.5 (6)	0.7 ± 0.8 (6)	0.5 ± 0.5 (6)	4 ± 8.4 (6)
	x	1 ± 1.7 (5)	0.5 ± 1 (4)	0.4 ± 0.9 (5)	0.2 ± 0.4 (6)
10	c	0.3 ± 0.5 (6)	2.3 ± 3.4 (6)	0.2 ± 0.4 (6)	8.2 ± 9.9 (5)
	x	0.3 ± 0.8 (6)	1.2 ± 2.9 (6)	3.5 ± 7.6 (6)	2.3 ± 2.7 (6)
11	c	0.7 ± 1.6 (6)	0.2 ± 0.4 (6)	0 ± 0 (5)	1.8 ± 1.7 (6)
	x	0.5 ± 1.2 (6)	0.3 ± 0.5 (6)	2.2 ± 1.8 (6)	2.5 ± 2.8 (6)

S2 Table D. Mean number of captured Acari individuals in 2015 across 4 treatment levels (control, water addition, moderate or severe drought). First table (Acari) includes all of the Acari specimens, whereas thereafter separate groups are shown. The rows contain the months and the previous treatments (C: control, X: extreme drought).

		**Acari (all groups)** (mean ± SD (number of samples))			
**month**	**first treatment**	**Control**	**Water**	**Moderate d.**	**Severe d.**
4	c	58.8 ± 44.6 (6)	45.2 ± 41.8 (6)	73.5 ± 34 (6)	75.7 ± 46.4 (6)
	x	44.5 ± 29 (6)	46.7 ± 30.8 (6)	73.3 ± 54.3 (6)	54.5 ± 61.7 (6)
5	c	41.7 ± 27.6 (6)	21.3 ± 19.5 (6)	62.3 ± 34.8 (6)	56.7 ± 18.8 (6)
	x	13.8 ± 10.7 (5)	15.3 ± 12.8 (6)	29.2 ± 31.4 (6)	21.7 ± 14.7 (6)
6	c	16.4 ± 19.1 (5)	5.2 ± 7.2 (6)	14.3 ± 10.9 (6)	41.4 ± 54.9 (5)
	x	12.5 ± 17.9 (4)	4.8 ± 7.1 (6)	12.8 ± 13.3 (5)	16.8 ± 22.8 (5)
7	c	29.5 ± 31.8 (2)	10.7 ± 18.5 (3)	10.3 ± 15.3 (4)	17 ± 17.6 (5)
	x	22 ± 28.8 (4)	12 ± 21.4 (4)	33.8 ± 52.4 (4)	20 ± 23 (5)
8	c	12 ± 10.1 (5)	1 ± 2 (4)	14.4 ± 16.7 (5)	33.8 ± 30.7 (6)
	x	8.5 ± 16 (6)	9 ± 15.3 (4)	27.7 ± 18.6 (3)	15.3 ± 14.9 (6)
9	c	15.5 ± 20.6 (6)	5.3 ± 9.8 (6)	20.5 ± 16.4 (6)	12.2 ± 6.9 (6)
	x	14.4 ± 18.8 (5)	14 ± 22.8 (4)	35.8 ± 62.4 (5)	4.8 ± 4.9 (6)
10	c	2.3 ± 3 (6)	3.2 ± 4.6 (6)	6.7 ± 7.6 (6)	12.8 ± 8.8 (5)
	x	3.3 ± 3.7 (6)	6.3 ± 8.7 (6)	9.8 ± 8.4 (6)	8.3 ± 5.9 (6)
11	c	0 ± 0 (6)	0 ± 0 (6)	2.6 ± 3.2 (5)	14.3 ± 32.2 (6)
	x	0.2 ± 0.4 (6)	1.8 ± 4 (6)	4.5 ± 5.5 (6)	15.5 ± 23.1 (6)

		**Astigmata** (mean ± SD (number of samples))			
**month**	**first treatment**	**Control**	**Water**	**Moderate d.**	**Severe d.**
4	c	1.7 ± 3.6 (6)	0.3 ± 0.5 (6)	6 ± 12.3 (6)	1.7 ± 3.6 (6)
	x	0.7 ± 1.6 (6)	0 ± 0 (6)	0 ± 0 (6)	0.2 ± 0.4 (6)
5	c	11.7 ± 16 (6)	2.7 ± 3.9 (6)	12.8 ± 16.1 (6)	4.5 ± 4.8 (6)
	x	0.2 ± 0.4 (5)	0.2 ± 0.4 (6)	0 ± 0 (6)	0.5 ± 0.5 (6)
6	c	6.2 ± 12.3 (5)	0.7 ± 1.2 (6)	3.5 ± 4.1 (6)	7 ± 8.2 (5)
	x	0.8 ± 1 (4)	0.2 ± 0.4 (6)	1.6 ± 2.3 (5)	8.2 ± 11.9 (5)
7	c	6 ± 8.5 (2)	2 ± 3.5 (3)	1 ± 1.4 (4)	4.6 ± 5.4 (5)
	x	8 ± 8.9 (4)	1.5 ± 1.9 (4)	2.3 ± 2.6 (4)	9.6 ± 11.4 (5)
8	c	1.6 ± 3.6 (5)	0 ± 0 (4)	3.8 ± 7.9 (5)	5.5 ± 3.9 (6)
	x	3 ± 5.9 (6)	5.5 ± 10.3 (4)	6.3 ± 5.7 (3)	4.3 ± 4.8 (6)
9	c	2.8 ± 4.2 (6)	1.5 ± 3.7 (6)	0.8 ± 1.2 (6)	1.7 ± 3.2 (6)
	x	1.2 ± 1.3 (5)	6 ± 10.1 (4)	28 ± 57.2 (5)	1.2 ± 1 (6)
10	c	0.7 ± 1 (6)	1 ± 1.5 (6)	0.2 ± 0.4 (6)	0 ± 0 (5)
	x	0.7 ± 0.8 (6)	0.5 ± 1.2 (6)	3.3 ± 4.4 (6)	1.3 ± 2.4 (6)
11	c	0 ± 0 (6)	0 ± 0 (6)	0.2 ± 0.4 (5)	0.3 ± 0.5 (6)
	x	0 ± 0 (6)	0 ± 0 (6)	1 ± 2.4 (6)	0 ± 0 (6)

		**Mesostigmata** (mean ± SD (number of samples))			
**month**	**first treatment**	**Control**	**Water**	**Moderate d.**	**Severe d.**
4	c	38.2 ± 34.9 (6)	31.8 ± 35.1 (6)	47.8 ± 29.2 (6)	55.7 ± 39.2 (6)

	x	25.7 ± 17.4 (6)	28.5 ± 17.1 (6)	57.5 ± 44.3 (6)	37.5 ± 50.9 (6)
5	c	24.8 ± 22.4 (6)	13.5 ± 14.5 (6)	30 ± 25.6 (6)	38.8 ± 19 (6)
	x	8.8 ± 6.6 (5)	10.7 ± 8.4 (6)	20 ± 20.7 (6)	10.3 ± 11.8 (6)
6	c	4.2 ± 3.5 (5)	1.8 ± 3.1 (6)	4.5 ± 5.1 (6)	28.4 ± 43.7 (5)
	x	8.8 ± 14.4 (4)	1.3 ± 2.3 (6)	4.8 ± 4.9 (5)	4.2 ± 6 (5)
7	c	9.5 ± 7.8 (2)	5.7 ± 9.8 (3)	4 ± 6.7 (4)	8.6 ± 9.6 (5)
	x	11.3 ± 19.9 (4)	8.8 ± 16.8 (4)	28.3 ± 47 (4)	10 ± 18 (5)
8	c	6.4 ± 5.5 (5)	0 ± 0 (4)	3.8 ± 5 (5)	14.7 ± 16.5 (6)
	x	4.5 ± 10.1 (6)	2.3 ± 3.2 (4)	15.3 ± 12 (3)	4.8 ± 4.4 (6)
9	c	6.5 ± 9.7 (6)	0.7 ± 1 (6)	5.2 ± 5.6 (6)	4.3 ± 3.5 (6)
	x	6.2 ± 10.1 (5)	3.8 ± 6.8 (4)	5.4 ± 5.5 (5)	2.2 ± 2.3 (6)
10	c	0.5 ± 0.8 (6)	0.5 ± 0.5 (6)	2.3 ± 3.4 (6)	7.2 ± 5.1 (5)
	x	1 ± 1.5 (6)	3.5 ± 5.4 (6)	3.3 ± 3.1 (6)	4.5 ± 3.3 (6)
11	c	0 ± 0 (6)	0 ± 0 (6)	1.8 ± 2.7 (5)	12.7 ± 29.1 (6)
	x	0.2 ± 0.4 (6)	0.8 ± 2 (6)	2.8 ± 3.1 (6)	13.7 ± 20.9 (6)

		**Oribatida** (mean ± SD (number of samples))			
**month**	**first treatment**	**Control**	**Water**	**Moderate d.**	**Severe d.**
4	c	4.2 ± 3 (6)	2.7 ± 2.3 (6)	3.7 ± 2.1 (6)	3.3 ± 1.6 (6)
	x	2.5 ± 1.9 (6)	2.8 ± 2.6 (6)	3.3 ± 3.2 (6)	2.8 ± 3.1 (6)
5	c	2.2 ± 2.5 (6)	2 ± 2.1 (6)	10.3 ± 6.3 (6)	6.5 ± 3.6 (6)
	x	1.6 ± 1.5 (5)	0.8 ± 1 (6)	2.3 ± 2.5 (6)	3.3 ± 2 (6)
6	c	3.4 ± 3.7 (5)	1.7 ± 2.9 (6)	2.5 ± 2.7 (6)	4.6 ± 4.3 (5)
	x	1.5 ± 2.4 (4)	1.7 ± 3.1 (6)	3.4 ± 4.1 (5)	3.4 ± 4.2 (5)
7	c	12 ± 15.6 (2)	1.7 ± 2.9 (3)	5.3 ± 7.5 (4)	2 ± 2.3 (5)
	x	1 ± 2 (4)	1.5 ± 2.4 (4)	2.8 ± 3.2 (4)	0.2 ± 0.4 (5)
8	c	3 ± 3.7 (5)	0.8 ± 1.5 (4)	5.4 ± 8 (5)	9.5 ± 9.2 (6)
	x	0.2 ± 0.4 (6)	0.3 ± 0.5 (4)	3.7 ± 0.6 (3)	3.7 ± 5.6 (6)
9	c	5.8 ± 8.2 (6)	2.8 ± 5.5 (6)	12 ± 10.2 (6)	5 ± 3.2 (6)
	x	6.2 ± 7.2 (5)	2.3 ± 3.9 (4)	1.4 ± 0.9 (5)	0.7 ± 1 (6)
10	c	0.8 ± 1.6 (6)	1.5 ± 2.8 (6)	3 ± 3.6 (6)	4.4 ± 4.3 (5)
	x	1.3 ± 1.8 (6)	1.8 ± 2.1 (6)	2.5 ± 2.7 (6)	1.2 ± 1.5 (6)
11	c	0 ± 0 (6)	0 ± 0 (6)	0.4 ± 0.5 (5)	0.7 ± 1.2 (6)
	x	0 ± 0 (6)	0.8 ± 1.6 (6)	0.2 ± 0.4 (6)	1.3 ± 2.4 (6)

		**Prostigmata** (mean ± SD (number of samples))			
**month**	**first treatment**	**Control**	**Water**	**Moderate d.**	**Severe d.**
4	c	14.8 ± 12.2 (6)	10.3 ± 9.4 (6)	16 ± 8.6 (6)	15 ± 11.5 (6)
	x	15.7 ± 12.6 (6)	15.3 ± 12.4 (6)	12.5 ± 9.2 (6)	14 ± 11.2 (6)
5	c	3 ± 3.9 (6)	3.2 ± 2.6 (6)	9.2 ± 10.2 (6)	6.8 ± 6 (6)
	x	3.2 ± 2.9 (5)	3.7 ± 4.9 (6)	6.8 ± 9.4 (6)	7.5 ± 8.5 (6)
6	c	2.6 ± 3.2 (5)	1 ± 1.7 (6)	3.8 ± 4.2 (6)	1.4 ± 0.9 (5)
	x	1.5 ± 1.9 (4)	1.7 ± 2 (6)	3 ± 3.5 (5)	1 ± 1.2 (5)
7	c	2 ± 0 (2)	1.3 ± 2.3 (3)	0 ± 0 (4)	1.8 ± 1.9 (5)
	x	1.8 ± 1.7 (4)	0.3 ± 0.5 (4)	0.5 ± 0.6 (4)	0.2 ± 0.4 (5)
8	c	1 ± 1.7 (5)	0.3 ± 0.5 (4)	1.4 ± 2.6 (5)	4.2 ± 5.7 (6)
	x	0.8 ± 1.3 (6)	1 ± 1.4 (4)	2.3 ± 4 (3)	2.5 ± 3.1 (6)

9	c	0.3 ± 0.8 (6)	0.3 ± 0.5 (6)	2.5 ± 5.2 (6)	1.2 ± 2.4 (6)
	x	0.8 ± 1.1 (5)	2 ± 2.2 (4)	1 ± 1.2 (5)	0.8 ± 1.6 (6)
10	c	0.3 ± 0.8 (6)	0.2 ± 0.4 (6)	1.2 ± 1.2 (6)	1.2 ± 1.6 (5)
	x	0.3 ± 0.5 (6)	0.5 ± 0.8 (6)	0.7 ± 0.5 (6)	1.3 ± 0.8 (6)
11	c	0 ± 0 (6)	0 ± 0 (6)	0.2 ± 0.4 (5)	0.7 ± 1.6 (6)
	x	0 ± 0 (6)	0.2 ± 0.4 (6)	0.5 ± 0.8 (6)	0.5 ± 0.8 (6)

S2 Table E. Mean (SD) values for the collembolan species in 2014. X: extreme drought treated, C: control sites.

Collembola species	C	X
**Most frequent species**	mean (SD) of individuals	
Arrhopalites caecus (Tullberg, 1871)	13.3 (42.2)	2.6 (9.8)
Entomobrya nigriventris Stach,1930	543.4 (928.3)	259.5 (257.5)
Proisotoma minuta (Tullberg, 1871)	88.2 (207.6)	7.1 (26.3)
Sphaeridia pumilis (Krausbauer, 1898)	45.4 (64.2)	0.5 (1)
**Rare species**		
Brachystomella curvula Gisin, 1948	1 (2.4)	0.3 (0.8)
Ceratophysella succinea (Gisin, 1949)		0.04 (0.2)
Cyphoderus albinus Nicolet, 1842	0.04 (0.2)	0.9 (3.2)
Entomobrya multifasciata (Tullberg, 1871)	0.04 (0.2)	0.3 (0.6)
Folsomia candida (Willem, 1902)		0.04 (0.2)
Heteromurus nitidus (Templeton, 1835)	0.04 (0.2)	
Lepidocyrtus cyaneus Tullberg, 1871	0.04 (0.2)	0.04 (0.2)
Orchesella taurica (Stach, 1960)		0.1 (0.3)
Protaphorura serbica (Loksa & Bogojevic, 1967)	0.1 (0.6)	
Sminthurus viridis (Linnaeus, 1758)	0.4 (0.8)	0.2 (0.5)
Sminthurus mulitpunctatus Schäffer, 1896	0.04 (0.2)	
Sminthurus maculatus Tömösváry, 1883	0.04 (0.2)	
Willowsia buski (Lubbock,1869)		0.1 (0.3)
Xenylla maritima Tullberg, 1869	0.2 (0.5)	0.2 (0.4)
