# Supplementary material for: Effects of single and repeated drought on soil microarthropods in a semi-arid ecosystem depend more on timing and duration than drought severity
Source: PLoS One. 2019 Jul 18;14(7):e0219975. doi: 10.1371/journal.pone.0219975 (PMC6638988; doi:10.1371/journal.pone.0219975)
Supplement: S3 File — (PDF) [file pone.0219975.s003.pdf]

Supplement 3

Introduction

The EDAPHOLOG monitoring system is a novel tool, which continuously detects microarthropods falling down into a trap containing an opto-electronic sensor and records the body sizes and the time of catching (Dombos et al., 2017). The new trap, which works with clay granules as medium between the soil and the trap, was previously compared with traditional sampling methods in a loamy soil. EDAPHOLOG caught more individuals of epedaphic species than soil extraction, but almost the same number of euedaphic microarthropods were found. Compared to pitfall traps, EDAPHOLOG probes caught lower number of epedaphic individuals, whereas euedaphic groups were also presented in the EDAPHOLOG samples (Dombos et al., 2017).

Unchanged attractiveness of the traps is an important requirement in experiments among different environmental circumstances. To identify the possible bias using EDAPHOLOG probes in sandy soils, we compared the differences among three sampling methods (pitfall trap, EDAPHOLOG trap, soil extraction). We hypothesize that the effectiveness of the 3 sampling methods differ from each other in the case of sandy soil similarly to the case of loamy soil (Dombos et al., 2017), and therefore, relative abundances of the animal groups will differ between the methods.

In addition, we also hypothesize that these differences in catchability will not be biased by climatic effects, such as drought treatment and irrigation. It is an important requirement of a trap used in climate change experiments. If catchability of the trapping methods does not change due to environmental conditions, we expect that the number of individuals from different mesofauna groups will have the same ratios under two different environmental conditions (humid and dry). Concerning soil extraction such bias cannot be expected.

Methods

Study site

The experiment took place in an open sand steppe (46°52’16.6”N, 19°25’17.7”E), near Fülöpháza in the Kiskunság National Park, in central Hungary, outside the fence of the droughts manipulation sites. The study site has sandy soil (calcaric arenosol) (Várallyay, 2005) with pH 7.8, 1.2% silt 1.5% clay and 97.3% sand content.

Sampling methods and experimental design

The study site was homogeneously covered by sand steppe, and it showed some spatial variability in plant species dominance. For that reason, within the site, we selected ten homogeneous plots, 3 x 3 m in size, which were internally homogeneous in vegetation. Two pitfall traps and two EDAPHOLOG traps were inserted into the soil of each plots (S3 Fig A). Climatic treatment and trapping began at 29. June 2018. As climatic treatments, 5 plots were irrigated (moisture treatment) and 5 plots were covered by polyethylene roofs (drought treatment; just the same way as it was used in the drought treatment of EXDRAIN project) (S3 Fig A). The experiment was conducted in a naturally dry period of the year, therefore, to generate differences in soil moisture, we had to add 10 l/ 9m^2 water by a sprinkler to the plots which were not covered by roof. Samples deriving from the two types of traps were collected at 17 July 2018. In addition, 2-2 soil cores were taken in a maximum half meter radius of the traps at the end of the experiment (S3 Fig A).

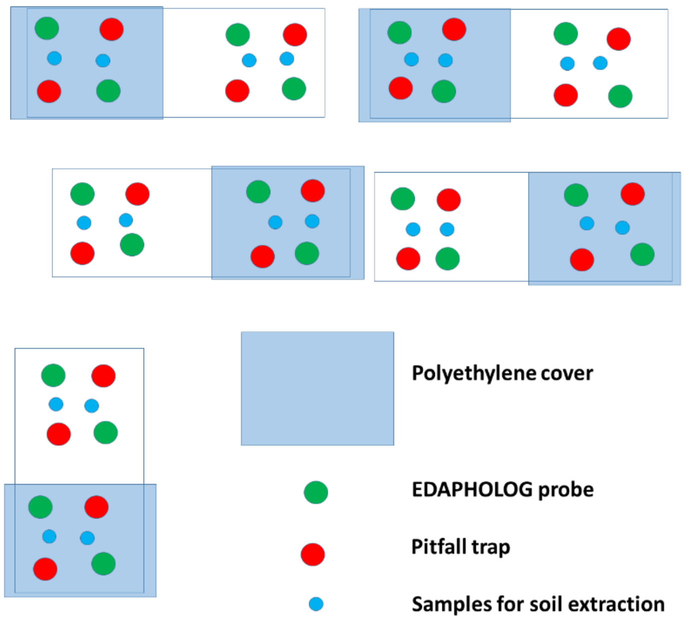

S3 Fig A. Experimental design of the10 plots. 5 plots for drought treatment were covered with a polyethylene roof to exclude precipitation and 5 plots for moisture treatment were irrigated.

The methods of trapping and sampling of mesofauna were the following:

I. Pitfall trap: 10 cm diameter funnel and a sample container (10-cm-diameter cup filled with preservative) for two weeks. Plastic plates were placed 1 cm above the funnels to prevent leaking.

II. EDAPHOLOG probes: commercially available horticultural clay granules were used as medium between soil and the probe, which collects animals from 0-10 cm upper part of the soil (S3 Fig B). In the middle, microarthropods vertically move through a perforated mesh tube (purple tube in S3 Fig B) and fall down and get caught. It was used to catch animals for two weeks and a plastic plate was used to prevent leaking.

III. Soil cores: soil cores with volume of 402.12 cm^3 (diameter: 8 cm, depth: 8 cm) were taken and extracted once at the end of the experiments.

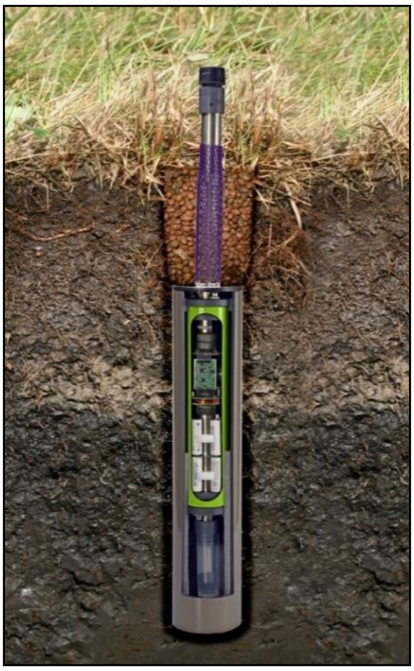

S3 Fig B. EDAPHOLOG probe with clay granule bag as matrix between the soil and the trapping system. The trapping tube is made by a purple net to prevent clay granules falling into the trap.

Sample proceeding

Samples of pitfall and EDAPHOLOG traps were stored in 70 % ethanol during the experiment. Soil cores were separately packed in plastic boxes and carried into the laboratory in 3 hours and extracted in a Berlese funnel extractor. Samples were stored in 70% ethanol.

Collembola were categorized into three groups, namely surface living, vegetation living and soil living Collembola. Mites were identified to and used in main groups (Astigmata, Mesostigmata, Oribatida, Prostigmata). The traps caught other invertebrates, not belonging to mesofauna, mainly macro-arthropods which were categorized into three groups: surface living macrofauna, soil living macrofauna and other invertebrates (S3 Table A).

S3 Table A. Number of individuals captured during the experiment. Codes of morphotypes: 1. soil living Collembola, 2. surface living Collembola, 3. vegetation living Collembola, 4. Mesostigmata, 5. Astigmata, 6. Prostigmata , 7. Oribatida, 8. soil living other mesofauna, 9. soil living not mesofauna, 10. surface living not mesofauna, 11. other invertebrates.

Categories	Morpho-types	Mean number (SD) of individuals captured by		
		soil extraction	pitfall	EDAPHOLOG
**Groups of mesofauna**				
surface living Collembola	1	1.7(1.2)	412.5(391.8)	71.4(155.5)
soil living Collembola	2	19.4(44.7)	661.7(1162.7)	8.5(11.0)
vegetation living Collembola	3	1.25(0.5)	4.6(4.1)	2.5(3)
Mesostigmata	4	13.1(25.2)	116.4(91.3)	9.9(36.6)
Astigmata	5	56.7(47.1)	9.3(12.1)	26.5(57.3)
Prostigmata	6	26(24.7)	95.2(60.5)	5.6(7.1)
Oribatida	7	21.6(29.3)	44.9(43.3)	3.3(3)
Diptera (larvae)	8	2.3(2.5)	6(9.1)	2.7(2.1)
**Groups not belonging to mesofauna**				
Coleoptera Staphylinidae	9	1(0)	3.4(3.1)	1.3(0.6)
Coleoptera Carabidae	10	1(0)	2.1(1.4)	1.5(0.6)
Coleoptera not Carabidae or Staphylinidae	10	1.1(0.4)	9.25(6.6)	1.25(0.5)
Coleoptera larvae	9	3.1(1.6)	2.8(1.6)	1.6(0.9)
Formicidae	10	6.25(5.9)	82.7(152.2)	22.6(31.0)
Hymenoptera, not Formicidae	11		21(14.2)	3(2.3)
Isopoda	9		1(0)	1(0)
Orthoptera	11		1.25(0.5)	
Psocoptera	11	1.8(1.5)	4.8(3.1)	1(0)
Hemiptera, not Cicada	11	2.8(2.2)	72.7(47.3)	17.8(42.9)
Cicada	11		13.3(23.9)	2(0)
Thysanoptera	9	1(0)	2.8(3.7)	1.5(0.7)
Diptera adult	11	1(0)	10.5(5.1)	3.8(2.9)
Araneae, 5 mm>	10	1(0)	7.7(8)	2(1.4)
Araneae, 5 mm<	10	1(0)	17.2(4.2)	1(0)
Lepidoptera	11		2.4(2.8)	1(0)
Mollusca	10	1(0)	2.3(2.3)	1(0)

Chilopoda, 5 mm<	9	1(0)
Diplopoda, 5 mm>	9	1(0)
Diplopoda, 5 mm<	9	1(0)
Neuroptera, Myrmeleontidae	9	1(0)

Environmental sampling

Soil moisture measurements were carried out at 29. June and 17. July 2018. Soil samples (two from each plots) were taken from the upper 10 cm of the soil to calculate soil moisture content (V/V%). Meanwhile soil temperature sensors (Decagon Devices 5TM) were inserted into the soil (10 cm depth), one in a drought and one in a water treated plot and were operated between 29 June and 17. July 2018. At the beginning, no difference occurred between the plots, soil humidity was low (S3 Fig C1). Irrigation significantly elevated soil water content by ca. 1.5 V/V% during the two-week long treatment (S3 Fig C1). These soil moisture contents are, however, very low values. The polyethylene cover increased the daily average temperature of the drought treated plots (S3 Fig C2). Daily total precipitation was derived from the nearest meteorological station, within 200 m (S3 Fig C3).

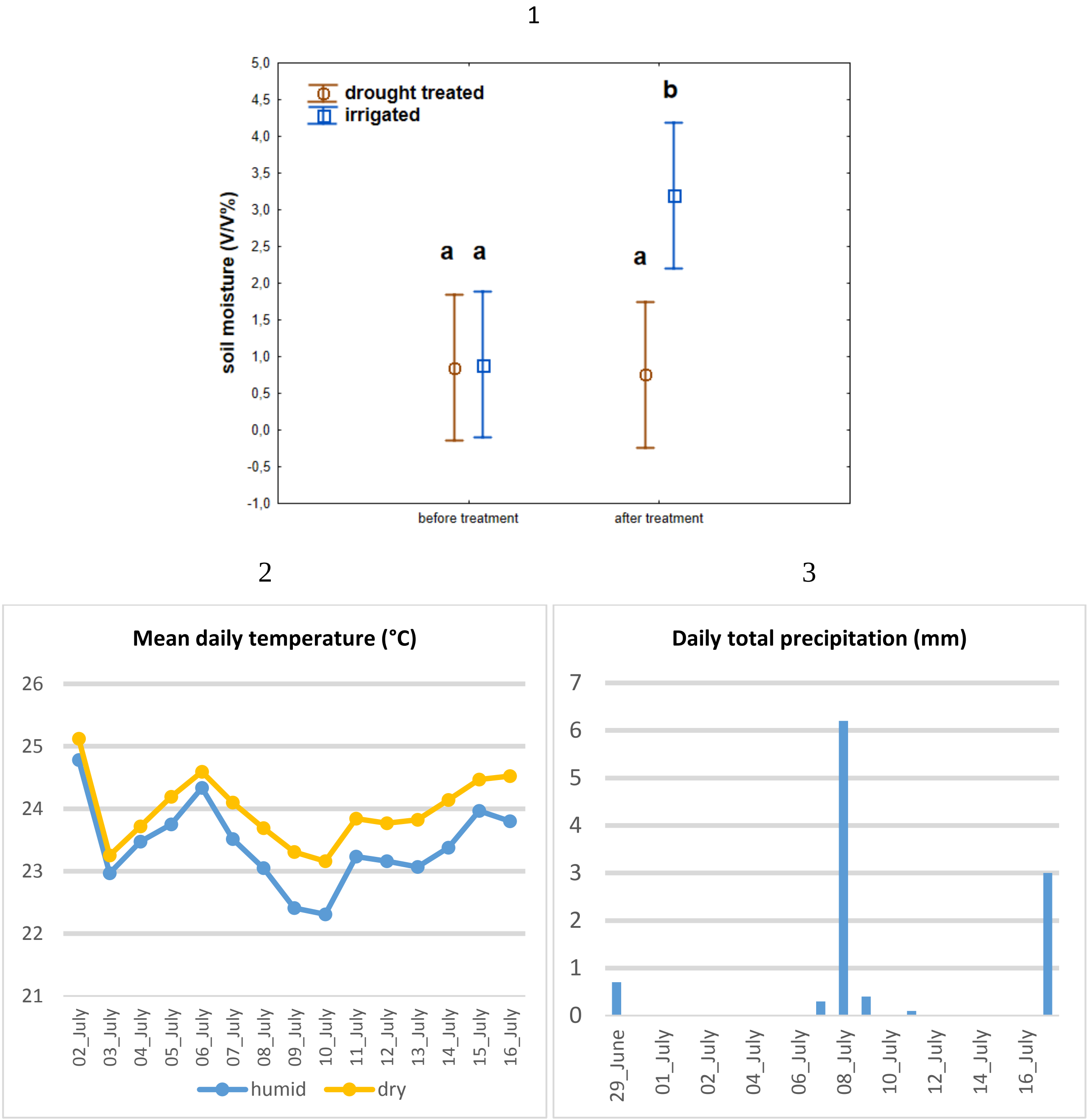

S3 Fig C. 1: Soil moisture contents of the plots (mean and SD, V/V%) (different letters show significant differences according to post-hoc tests of two-way MANOVA . 2: mean daily temperature of an irrigated and moisture experimental plots experimental plots, and 3: daily total precipitation data of the nearest meteorological station.

Statistical Analysis

To test the difference in the number of capture efficiency and the effects of environmental humidity between the three types of sampling methods, number of individuals captured by 20 EDAPHOLOG and 20 pitfall traps and 20 extracted soil cores were available for the analyses. To test the difference in the number of captured individuals (from 11 animal

groups) between the three methods and the two different conditions of humidity, a two-way MANOVA model was built (STATISTICA software). The response variables were the numbers of individuals of the above mentioned animal groups, whereas the explanatory factors were the three methods (soil cores, EDAPHOLOG, pitfall traps) and the two levels of humidity (dry, humid). For the analyses, the response variables were ln(x+1) transformed to eliminate heteroscedasticity and skew of the residuals.

Results

In the area during the experiment, traps collected 35743 animals, from that 31602 belonged to mesofauna (S3 Table A). According to the two-way MANOVA, only the effects of the sampling method were significant (f2). In case of the state of humidity (f1) and the interaction between f1 and f2, no significant result was found (MANOVA, f1: Wilk's $\lambda = 0.025$, $F_{22,88} = 21.37$, $p < 0.001$; f2: Wilk's $\lambda = 0.749$, $F_{11,44} = 1.34$, $p = 0.237$; f1*f2: Wilk's $\lambda = 0.671$, $F_{11,65} = 0.88$, $p = 0.614$).

Follow-up ANOVA models gave different results for animal groups (S3 Table B). Following Bonferroni's Type I error correction, there was no significant difference found in different soil humidity conditions, and there was no significant interaction between sampling methods and the soil humidity state in case of any animal groups.

Pitfall traps caught more individuals than the other two methods (S3 Fig D). However, these samples contained many invertebrates not belonging to mesofauna. Pitfall traps were most efficient in capturing all Collembolan groups, mesostigmatid, oribatid and prostigmatid mites, other mesofauna, surface and soil living macrofauna. The results were not so pronounced between the three traps in case of astigmatid mites and the "other" macrofauna group. Among the three methods, soil cores included the lowest number of individuals in case of surface living Collembola (S3 Fig D). In sandy soil, EDAPHOLOG was less efficient in case of prostigmatid and oribatid mites. Soil extraction – in contrast to pitfall trap and EDAPHOLOG – provides the density of mesofauna in the sampling time.

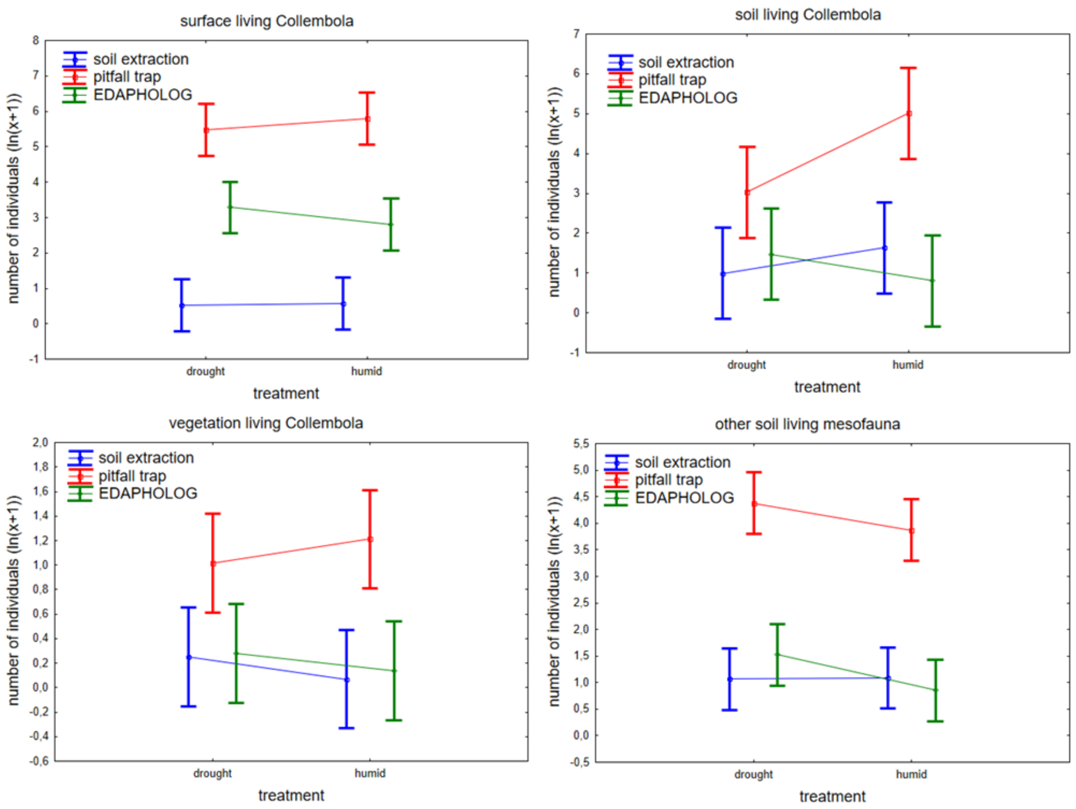
surface living Collembola
soil living Collembola
vegetation living Collembola
other soil living mesofauna
soil extraction
pitfall trap
EDAPHOLOG
number of individuals (ln(x+1))
drought
humid
treatment

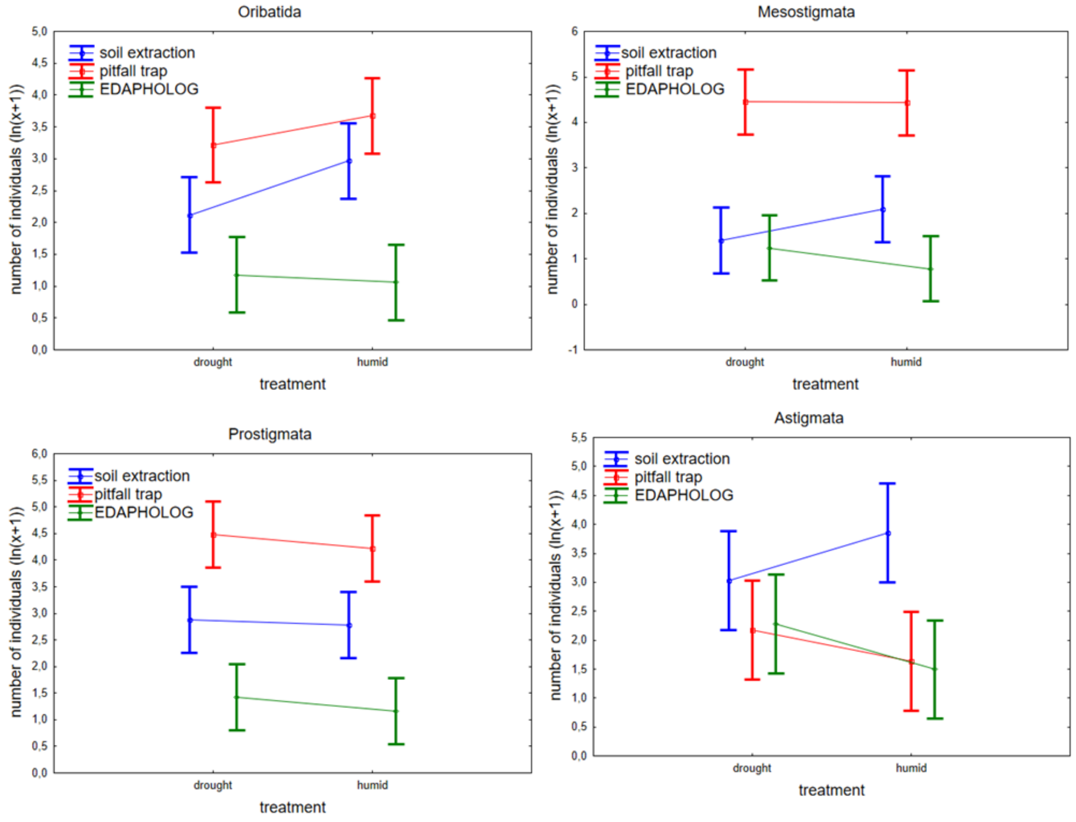
Oribatida
Mesostigmata
Prostigmata
Astigmata
soil extraction
pitfall trap
EDAPHOLOG
number of individuals (ln(x+1))
drought
humid
treatment

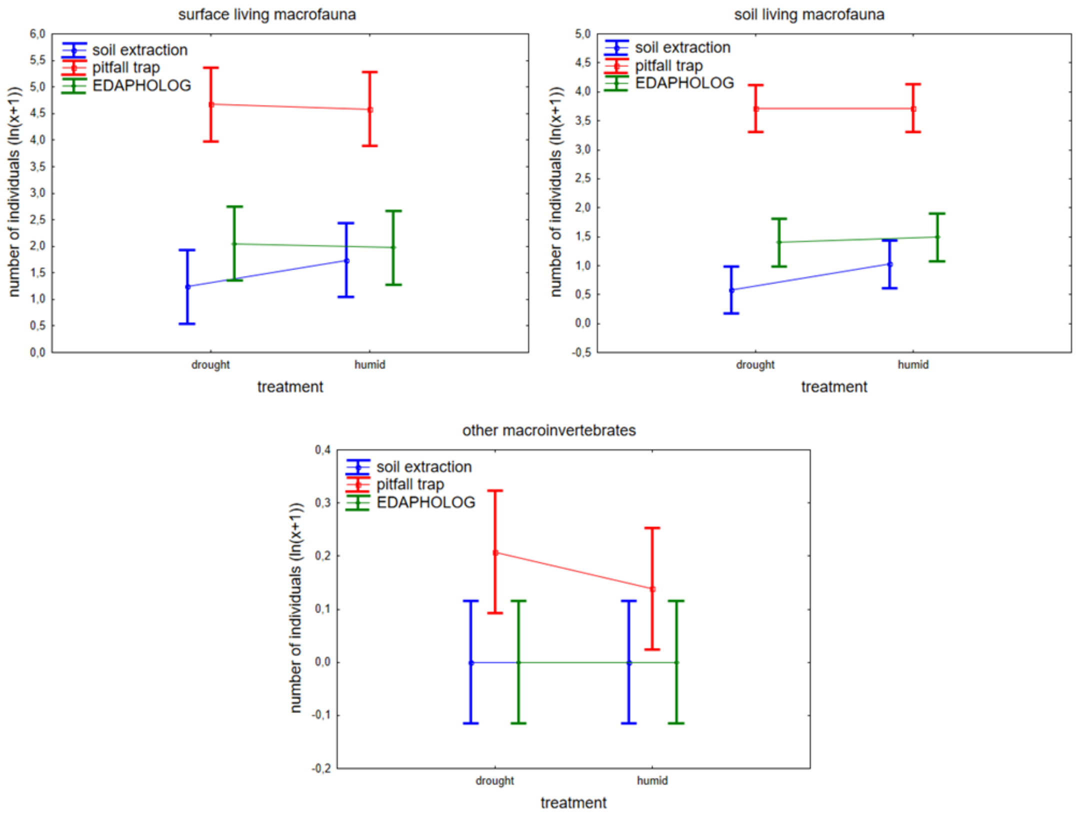

S3 Fig D. Mean number of individuals (with a confidence interval of 95%) of the 11 animal groups captured by three methods (EDAPHOLOG, pitfall traps, soil extraction) in two different humidity conditions (humid, dry).

S3 Table B. The effect of humidity on the catching efficiency of different traps (MANOVA). f1: trapping methods (EDAPHOLOG, pitfall trap, soil extraction), f2: humidity state (dry, humid period). “n.s.” indicates the case when the significance level was higher than p=0.1 after Bonferroni’s Type I error correction.

group	factor	$F_{22, 88}$	p
surface living Collembola	**f1**	**97.645**	**<0.001**
	f2	0.019	n.s.
	f1*f2	0.615	n.s.
soil living Collembola	**f1**	**16.022**	**<0.001**
	f2	1.975	n.s.
	f1*f2	2.697	n.s.
vegetation living Collembola	**f1**	**14.319**	**<0.001**
	f2	0.064	n.s.
	f1*f2	0.517	n.s.
Mesostigmata	**f1**	**50.810**	**<0.001**
	f2	0.055	n.s.
	f1*f2	1.281	n.s.
Prostigmata	**f1**	**49.060**	**<0.001**
	f2	0.690	n.s.
	f1*f2	0.045	n.s.
Astigmata	**f1**	**8.801**	**<0.001**
	f2	0.235	n.s.
	f1*f2	2.092	n.s.
Oribatida	**f1**	**31.605**	**<0.001**
	f2	2.716	n.s.
	f1*f2	1.353	n.s.
other soil living mesofauna	**f1**	**71.755**	**<0.001**
	f2	2.693	n.s.
	f1*f2	0.794	n.s
soil living macrofauna	**f1**	**112.003**	**<0.001**
	f2	1.151	n.s.
	f1*f2	0.664	n.s.
surface living macrofauna	**f1**	**46.967**	**<0.001**
	f2	0.147	n.s.
	f1*f2	0.471	n.s.
other macrofauna	**f1**	**6.081**	**<0.05**
	f2	0.244	n.s.
	f1*f2	0.243	n.s.

Discussion

The purpose of the experiment was to evaluate the catching efficiency of EDAPHOLOG probes in sandy soil with comparing them to commonly used and accepted sampling methods (soil extraction and pitfall trap). The other important aspect was whether this catching efficiency was modified by different climatic conditions (drought and humid circumstances).

We found significant differences in the catching efficiencies of the three sampling methods for different animal groups. In sandy soil, using pitfall traps seems to be more effective than other sampling methods for several animal groups. EDAPHOLOG usually did not differ significantly from the catching efficiency of soil extraction. In case of surface living Collembola, EDAPHOLOG showed an intermediate catching efficiency between the two other methods. For the other mesofauna groups, we conclude that the capture efficiency of EDAPHOLOG probes was weaker in sandy soils than in loamy soils (Dombos et al., 2017). It was especially true for oribatid and prostigmatid mites, which were gained in the lowest number by EDAPHOLOG probes.

Soil extraction is especially difficult in dry sand soils where extraction may not work properly and that may also influence the results. For such loose soil types, floating technique (adding olive oil to samples) is considered to be an effective method to divide animals from soil particles making animals floating on the top of liquid (Kuenen et al., 2009). Besides this method, in sandy soils, the trap part of EDAPHOLOG probes may have good potentials, because it provides an alternative of other sampling methods, which are difficult to use in sandy soil (see below). However, it seems that EDAPHOLOG probes perform better catching efficiencies for motile organisms such as surface living Collembola. Animals have to move vertically to be trapped among the clay granules. For future investigations, we suggest, that by reducing the amount of clay granules around traps, sampling of soil microarthropod groups with lower motility would be more effective.

Results of soil extraction, which in many soil types is considered to be one of the best estimation methods for soil microarthropod density, did not significantly differ between the two climatic manipulations (drought treatment and irrigation), and that was reflected in activity density data gained by pitfall traps and EDAPHOLOG probes, as well. EDAPHOLOG caught few animals, even compared to soil extraction method. This may be derived from the fact that the experiment was conducted in a naturally dry period and for a short time (only for 2 weeks). However, in spite of the low number of animals found in EDAPHOLOG traps, the rates of animals caught in different conditions did not significantly differ from the other conventional methods. Moreover, in this sandy soil, pitfall traps and soil extraction provided very dirty

samples (samples contained soil particles and plant remains), from which sorting of animals was difficult and time consuming. By contrast, EDAPHOLOG probes provided clean samples with lower number of animals, and that has advantages in a long term. We conclude that EDAPHOLOG is applicable in climate manipulation experiments, without continuously disturbing the environment and slightly influencing natural populations of the area even for a long term.

References

Dombos M, Kosztolányi A, Szlávecz K, Gedeon Cs, Flórián N, Groó Z, et al. 2017. EDAPHOLOG monitoring system: automatic, real-time detection of soil microarthropods. Methods in Ecology and Evolution 8: 313-321.

Kuenen FJ, Venema H, van Gestel CA, Verhoef HA (2009) Extracting soil microarthropods with olive oil: A novel mechanical extraction method for mesofauna from sandy soils. European Journal of Soil Biology 45: 496-500.

Várallyay Gy (2005) Magyarország talajainak vízraktározó képessége. Agrokémia és Talajtan 54: 5-24.
