# Supplementary material for: Effects of single and repeated drought on soil microarthropods in a semi-arid ecosystem depend more on timing and duration than drought severity
Source: PLoS One. 2019 Jul 18;14(7):e0219975. doi: 10.1371/journal.pone.0219975 (PMC6638988; doi:10.1371/journal.pone.0219975)
Supplement: S4 File — (PDF) [file pone.0219975.s004.pdf]

## Supplement 4

In our study, we focused on changes of mesofauna assemblages, however, it is worth to mention some species-level changes, because ecological habits of dominant species could explain some observed patterns.

Species from the area, like *Entomobrya nigriventris* are usually found in dry environments, however, their response to drought is still unknown. They can be considered as xerothermophil, surface-active species, with a high moving capacity. Therefore, they might survive the dry periods by horizontal migration and by finding refuges [1]. This could be the reason of their AD decline in moderate and severe drought treatments. As an immediate response, they could emigrate from the plot during treatment and immigrate when the conditions became favourable. In this article, eu- and hemiedaphic Collembola species were considered as soil living Collembola group. Based on Salmon et al. [2], these two groups considering their habitat requirements are statistically close to each other and to those species, which are living in closed habitats. Compared to surface living Collembola species, they live among more stable habitat, and did not get used to rapid changes of environmental factors. Although, they can migrate vertically in the soil and create aggregates [1], they are sensitive to environmental changes. Despite the fact that hemi- or euedaphic species (such as *Xenylla maritima* and *Proisotoma minuta*, which are present with the highest AD) found in the area are considered to be drought tolerant, against an extreme, long drought period, when desiccation reach the deeper soil layers, they are exposed and unprotected. This could be the reason why we found decrease in their AD during extreme drought. The dominant species in the vegetation living Collembola group was *Sphaeridia pumilis*. Activity density of this species predominantly influenced the reaction of the vegetation living Collembola group. In the literature there are contrary information about the traits of this animal. Bretfeld and Dunger [3] mention *S. pumilis*

as vegetation living species, according to Gisin [4] and Ponge [5], it is hemiedaphic, whereas it is even considered to be euedaphic [6]. We followed the categorization of Salmon et al. [2], which was based on statistical methods considering vegetation living and morphological traits (pigmentation, number of eyes, and the developed furcula). We suspect, that after the death of vegetation, this species is able to feed on and live in the leaf litter and can be considered as hemiedaphic species, which in our case could influence the effect of extreme drought on soil living Collembola groups even more evidently. But the results of the second treatment (2015) would not be affected.

Activity density of mite groups were higher in extreme drought treatments compared to control plots. Acari were reported to cope better with drought [7,8] or to react negatively to elevated water content of soil [9,10]. The xerophil *Scutovertex sculptus* and *Passalozetes perforatus* were the outstanding most frequent species which were found to be dominant in the study site [11]. Compared to Collembola, due to their strong cuticle, mites are considered to be more resistant [12] and may adapt better to higher environmental fluctuations [7], and they may remain active for a longer time among dry conditions. In addition, high amount of dead plant material in drought treatment may have been an important food source for primary and secondary decomposer mites like the fungal feeding *Passalozetes perforatus* [13].


1. Hopkin SP (1997) Biology of the springtails:(Insecta: Collembola): OUP Oxford.
2. Salmon S, Ponge J-F, Gachet S, Deharveng L, Lefebvre N, Delabrosse F (2014) Linking species, traits and habitat characteristics of Collembola at European scale. Soil Biology and Biochemistry 75: 73-85.
3. Bretfeld G, Dunger W (1999) Synopses on Palaearctic Collembola: Symphypleona: Staatlicjes Museum für Naturkunde Görlitz.
4. Gisin H (1943) Ökologie und Lebensgemeinschaften der Collembolen im schweizerischen Exkursionsgebiet Basels: Inauguraldissertation... vorgelegt der philosophisch-naturwissenschaftlichen Fakultät der Universität Basel von Hermann Gisin: A. Kundig.
5. Ponge J-F (1993) Biocenoses of Collembola in atlantic temperate grass-woodland ecosystems. Pedobiologia 37: 223-244.
6. Szeptycki A (1967) Fauna of the springtails (Collembola) of the Ojców National Park in Poland: Zakład Zoologii Systematycznej Polskiej Akademii Nauk.
7. Kardol P, Reynolds WN, Norby RJ, Classen AT (2011) Climate change effects on soil microarthropod abundance and community structure. Applied Soil Ecology 47: 37-44.

8. Vestergård M, Dyrnum K, Michelsen A, Damgaard C, Holmstrup M (2015) Long-term multifactorial climate change impacts on mesofaunal biomass and nitrogen content. Applied Soil Ecology 92: 54-63.
9. O'Lear H, Blair J (1999) Responses of soil microarthropods to changes in soil water availability in tallgrass prairie. Biology and Fertility of Soils 29: 207-217.
10. Liu J-L, Li F-R, Liu L-L, Yang K (2017) Responses of different Collembola and mite taxa to experimental rain pulses in an arid ecosystem. Catena 155: 53-61.
11. Mahunka S (1987) A survey of the oribatids of the Kiskunság National Park (Acari: Oribatida). The fauna of the Kiskunság National Park 2: 346-397.
12. Schaefer M (1995) Interspecific interactions in the soil community. Acta Zoologica Fennica: 101-106.
13. Schatz H (1983) Catalogus Faunae Austriae. Teil IX U Ordn: Oribatei, Hornmilben–Österreichische Akademie der Wissenschaft, Vienna.
